# Molecular level study of hot water extracted green tea buried in soils - a proxy for labile soil organic matter

**DOI:** 10.1038/s41598-020-58325-8

**Published:** 2020-01-30

**Authors:** Nicholle G. A. Bell, Alan J. Smith, Yufan Zhu, William H. Beishuizen, Kangwei Chen, Dan Forster, Yiran Ji, Elizabeth A. Knox

**Affiliations:** EaStChem, School of Chemistry, University of Edinburgh, David Brewster Rd, Edinburgh, EH9 3FJ UK

**Keywords:** Carbon cycle, Environmental monitoring, Mass spectrometry, Solution-state NMR, Cheminformatics

## Abstract

Understanding the composition of soil organic matter (SOM) is vital to our understanding of how soils form, evolve and respond to external stimuli. The shear complexity of SOM, an inseparable mixture of thousands of compounds hinders the determination of structure-function relationships required to explore these processes on a molecular level. Litter bags and soil hot water extracts (HWE) have frequently been used to study the transformation of labile SOM, however these are still too complex to examine beyond compound classes. In this work, a much simpler mixture, HWE buried green tea, was investigated by Nuclear Magnetic Resonance (NMR) spectroscopy and Fourier Transform Ion Cyclotron Resonance Mass Spectrometry (FT-ICR-MS), as a proxy for labile SOM. Changes induced by the burial over 90 days in a grassland, woodland and two peatland sites, one damaged by drainage and one undergoing restoration by drain-blocking, were analysed. Major differences between the extracts were observed on the level of compound classes, molecular formulae and specific molecules. The causes of these differences are discussed with reference to abiotic and biotic processes. Despite the vastly different detection limits of NMR and MS, chemometric analysis of the data yielded identical separation of the samples. These findings provide a basis for the molecular level interrogation of labile SOM and C-cycling processes in soils.

## Introduction

Earth’s soils store a greater amount of C than both the vegetation and atmosphere combined and thus play a crucial role in the global C cycle^1^. The accumulation of C is largely the result of restricted decomposition rates, which are ecosystem dependent. Three major causes for these restrictions include climate (temperature and moisture), substrate quality (chemical and physical characteristics), and the composition, abundance and activity of the soil biotia^2,3^.

Soil decomposition has historically been studied using litter bags, which consist of plant material of known mass enclosed in a screened container^4,5^. A variety of different litter bag compositions and protocols have been used over the years, which makes a cross-study comparison practically impossible^6,7^. More recently, a standardized method using green and rooibos Lipton tea bags has emerged^8^, which involves burying pairs of pre-weighed green and rooibos tea bags 15 cm apart to a depth of 8 cm. After 90 days, the tea bags are collected, dried and weighed. Using the fact that the rooibos tea decomposes slower than the green tea permits the so-called stabilization factor, *S* and decomposition rate, *k*, to be calculated from the determined weight losses. These two variables constitute the Tea Bag Index (TBI) which has been measured for different soils worldwide in order to define the relationships between soil types, decomposition rates and environmental factors^6,8–10^. The tea bag decomposition rates, *k*, have been shown to positively correlate with those of local litter and respond in similar ways to biotic factors^7,9,11^.

On a molecular level soil litter decomposition can be divided into, at least, two stages^12^. The first stage involves the non-lignin and soluble compounds, e.g. carbohydrates, small phenolics, amino acids, which are easily leached, hydrolysed or oxidised, while the second stage involves the transformation of recalcitrant molecules^6^, e.g. lignin-like compounds. Considering a burial period of 90 days and the typical turnover rates of different litter compound classes^13^, the latter stage will not occur to a significant extent^8^ in soils of temperate climates, therefore largely the labile SOM is affected on this time scale. Labile SOM is considered to be the most sensitive indicator of SOM quality and decomposability^14,15^, which justifies the study of this soil pool.

Green tea is produced from the same tea leaves as black tea (*Camellia sinensis*) with the only difference being the lack of an oxidation step in the manufacturing process. Rooibos tea is produced from the leaves and stems of the *Aspalathus linearis* bush, which are oxidized^16^. The addition of stems as well as the oxidation process make rooibos tea less labile (less soluble)^7,8,17^, and thus less suitable for monitoring the initial stages of decomposition over the 90 day TBI burial period.

The composition of green tea has been studied extensively and it is known to consist of a mixture of phenolics, terpenoids, xanthines, alkaloids, flavones, flavonoids, fatty acids, amino acids and sugars^18–21^, with each compound class containing many molecules. For example Hooft *et al*. produced the most comprehensive list to date of tea phenolics identifying a total of 177 compounds^22^. Of these, polyphenolics called catechins exist in the highest concentration dominated by epigallocatechin gallate (EGCG) followed by epigallocatin (EGC)^23^.

Undoubtedly simpler than the heterogeneous plant litter or SOM itself, monitoring the changes of buried tea on the molecular level is therefore feasible and could advance our understanding of decompositional processes that occur in soils. To study these processes, some form of OM isolation is required. Considering that hot water extracted (HWE) SOM is widely regarded as representative of labile SOM^15,24,25^, buried tea was subjected to HWE in this study. Unlike cold water extraction, HWE yields several times the amount of C and also extracts microbial metabolites and rhizodeposits^15,26^. It therefore represents both the labile OM and microbial biomass.

Solubilised tea OM is a complex mixture that requires powerful analytical techniques, liquid-state NMR and FT-ICR-MS^27,28^, to be studied. This study presents molecular level compositional differences of HWE buried green tea as a proxy for labile SOM decomposition of three different soil types: species rich grassland, woodland and peatland.

## Results

### NMR analysis of Hot Water Extracted (HWE) green tea samples

**Figure 1:**
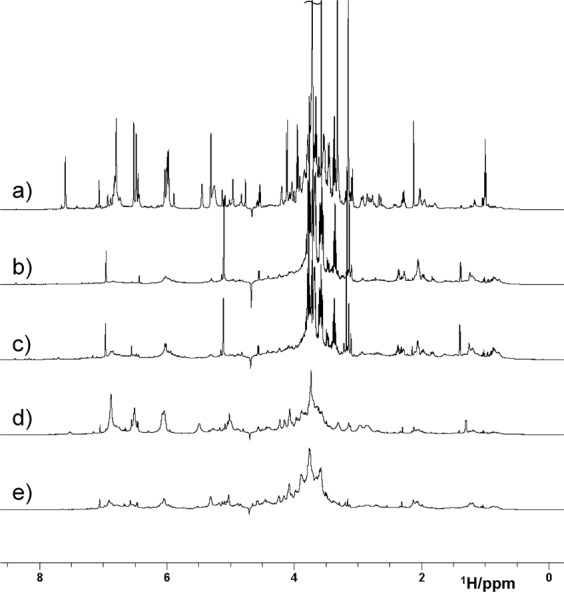
600 MHz ^1^H NMR spectra of the HWE (**a**) unburied, (**b**) grassland, (**c**) woodland, (**d**) RMII and (**e**) RMI buried green tea. The assignment of the unburied tea signals is given in Supplementary Fig. S2.

An overlay of representative 1D ^1^H spectra from HWE buried and buried tea is shown in Fig. 1.

The spectra can be divided into three main regions: 8.5–6 ppm – aromatics (mostly catechins), caffeine (in the unburied tea); 5.5–3 ppm – mostly carbohydrates, caffeine, amino acids, catechins and organic acids; and 3–0 ppm, aliphatic molecules and moieties, such as amino acids, fatty acids and catechins. Assignment of the major signals present in the ^1^H NMR spectrum of the HWE unburied tea based on literature data^22,29,30^ and 2D NMR analysis^31^, is given in Supplementary Fig. S2. The spectrum of unburied green tea shows a number of sharp resonances, as one would expect for a mixture of small to medium size molecules. Signals of a polysaccharide, amylopectin^32^, were also identified (Supplementary Fig. S3). There are clear differences between the HWE spectra of the buried and unburied tea, which indicate that burial has changed the molecular composition. Starting with the aromatic region, the signals of catechins dominate the spectra obtained from the unburied tea and the Red Moss of Balerno raised bog under restoration (herein referred to as RMII), as broad signals in the latter sample, while these are greatly reduced in the other samples (Fig. 1 and Supplementary S4). The carbohydrate region separates spectra into two groups: peatland and grassland/woodland samples. For both groups, most of the unburied tea carbohydrates have disappeared (Supplementary Figs. S5 and 6), including the polysaccharide, amylopectin. New polysaccharide signals appeared in the spectra of peatland samples that belong to arabinogalactans - a known component of the green tea^33^. While in the woodland/grassland samples the small carbohydrates, trehalose and mannitol were identified, which are known stress molecules of microbial origin (Supplementary Fig. S7)^34,35^. Finally, in the unburied tea, the region below 3 ppm contains signals of typical tea compounds (CH_2_ groups of catechins, theanine - a tea specific amino acid -, other amino acids, carboxylic acids and fatty acids). In the peatland samples these CH_2_ signals are broad with additional broad humps appearing at around 2.1, 1.3 and 0.9 ppm in all four samples, again with distinct differences between the peatland samples and the grassland/woodland samples. The signals of theanine (and also caffeine) disappeared in all samples and compounds such as alanine (1.47, 3.76 ppm) became more pronounced in the spectra of the latter two samples, while lactic acid (1.32, 4.1 ppm) appeared strongly in the grassland samples. Several grassland samples also contained elevated levels of fatty acids. Some of the spectra obtained from the damaged Red Moss of Balerno raised bog site (herein referred to as RMI) were similar to the RMII spectra, while the others appeared similar to the woodland spectra. The characteristics described above are typical for the majority of the spectra obtained from an individual site (Supplementary Figs. S8–S11).

### Relaxation and Diffusion Order SpectroscopY (DOSY) analysis of HWE green tea samples

To investigate the nature of the changes in the ^1^H spectra discussed above, *T*_1_ and *T*_2_ relaxation and DOSY experiments were recorded on the representative samples presented in Fig. 1. Generally, the *T*_1_ and *T*_2_ relaxation times of buried samples shortened significantly (see Supplementary Tables SIII–SV and Figs. S12–S21) relative to the unburied tea sample. The RMI sample had the shortest relaxation times, while the woodland sample had values close to the unburied tea sample. These experiments indicated that, within the NMR detection limits, both large and small molecules were generated by the burial process, unexpectedly across the compound class range.

**Figure 2:**
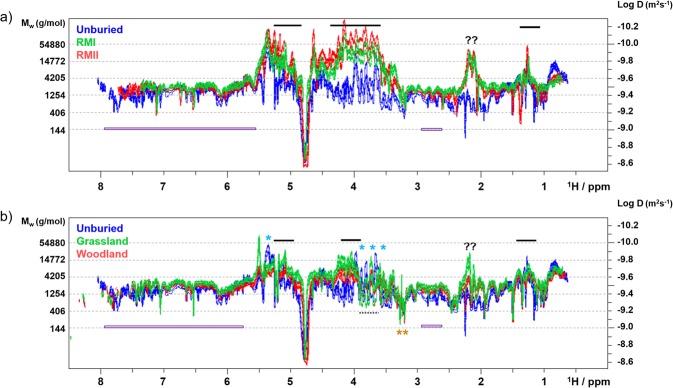
The 800 MHz 2D DOSY spectra of the HWE tea samples. Overlay of the (**a**) unburied tea spectra with RMI/RMII and (**b**) grassland/woodland spectra. Selected areas are labelled and discussed in the text. Blue stars above the spectra indicate signals of amylopectin, arabinogalactans are labelled by horizontal bars, dashed bars indicate signals of trehalose and mannitol. Orange stars indicate OCH_3_ groups. Question marks label unassigned signals from large molecules. Catechin signals are marked by purple bars. The shift of the signals towards larger molecular weight seen in the grassland spectra could be due to presence of oxidation products of catechins, e.g. Theasinensins^42^.

Analysis of the DOSY spectra (Fig. 2) confirmed the results of the relaxation experiments showing a spread of molecular sizes between 90 g/mol to 55,000 g/mol (Supplementary Fig. S22). The DOSY peaks of the unburied tea sample appeared in the expected molecular range, reflecting the presence of small (amino acids such as theanine, caffeine, theobromine, gallic acid, glucose), medium (sucrose, catechins) and large (mainly amylopectin) molecules.

The DOSY spectrum of the RMII sample confirmed that the broad signals in the aromatic region belong to catechins and not to large molecules. The broadness thus being a consequence of heterogeneity due to their partial modifications or dynamic aggregation. The dominant and unique feature of the DOSY spectra of the RM samples (Fig. 2, Supplementary Figs. S24 and 25) is the appearance of a large arabinogalactan (Supplementary Figs. S5 and S6)^33^. On the other hand, sharp and intense signals resonating between 3.5 and 4 ppm in the spectra of the woodland and grassland samples (Supplementary Figs. S26 and 27) belong to trehalose and mannitol^34,35^.

### Chemometric analysis of 1H NMR spectra of HWE green tea samples

**Figure 3:**
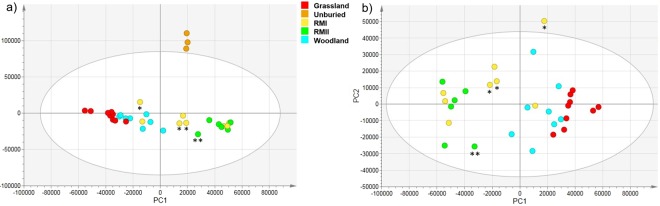
PCA score plot based on the 1D ^1^H 600 MHz NMR spectra of (**a**) all 34 green tea samples: unburied, tea buried in grassland, woodland, RMI and RMII, (**b**) only buried tea samples. The legend indicates the colour coding for each sample site. The Hotelling’s T^2^ ellipse represents the 95% confidence interval. Outlying RMI or RMII samples are marked by a single or double asterisks, respectively.

Principal Component Analysis (PCA) of ^1^H spectra of 34 HWE tea samples was performed to characterise the sample set. Two principle components were extracted (Fig. 3a) accounting for 76% of the total variance (39 and 37% by PC1 and PC2, respectively). The percentage of the total predicted variance was 85% (28 and 57% by PC1 and PC2, respectively). As expected, the unburied tea samples clearly separated from the rest, falling outwith the 95% Hotelling’s T^2^ ellipse.

Focusing on the buried samples, PCA was repeated excluding spectra of the unburied tea (Fig. 3b). Two principle components were again extracted accounting for 72% of the total variance (59 and 12% by PC1 and PC2, respectively). The percentage of the total predicted variance was 75% (56 and 20% by PC1 and PC2, respectively). The samples separated along PC1 in a similar manner as seen in the relaxation and DOSY experiments, into RMI/RMII and grassland/woodland groups. The largest separation was seen between the RMII and grassland samples, while the woodland samples partially overlapped with the grassland samples. The RMI samples were the most heterogeneous group, with some overlapping with the RMII and others with the woodland samples. Visual inspection of the RMI ^1^H NMR spectra confirmed that the respective samples share a similar appearance to either the RMII or woodland spectra (Supplementary Figs. S10 and S11). Overall, the PCA analysis of the ^1^H NMR spectra of the HWE buried tea indicated that the within-group variation was significantly less than the between-group variation for the RMII and grassland samples. The PC1 loadings (Supplementary Fig. S28) identified spectral regions responsible for this separation reflecting the disappearance of catechins and the appearance of trehalose, mannitol and aliphatic compounds (unassigned signals at ~3.15 ppm) in the woodland, grassland and some RMI samples, as well as the appearance of arabinogalactans in most RMII and some RMI samples. The amylopectin anomeric signals visible in the PC2 loadings at ~5.4 ppm were most abundant in some of the RMI spectra.

The subsequent PLS-DA analysis (Supplementary Figs. S29 and 30), in addition to demonstrating a clear separation of the RMII and grassland samples seen by PCA, also separated the woodland samples from the rest. Some RMI samples continued to overlap with RMII samples, while some moved towards the woodland samples. The regions of the NMR spectra that contributed most to the PLS clustering are indicated in the form of biplots (Supplementary Fig. S29); the compounds resonating in these areas were identical to those identified by PCA analysis presented above. The hierarchical clustering analysis (HCA) dendrogram based on PLS-DA (Supplementary Fig. S30) mirrors the clustering of the PLS-DA score plots with labelling of individual samples linking them to the original ^1^H NMR spectra (Supplementary Figs. S8–11).

Despite significant overlap of ^1^H signals, chemometric analysis of the NMR data identified differences between the composition of the HWE buried tea from different soil types and provided structures of discriminatory molecules.

### FT-ICR-MS analysis of the HWE green tea samples

The sensitivity and resolution of FT-ICR-MS provides a complementary view to the analysis of tea offered by NMR. High quality MS spectra were obtained for unburied and buried green tea samples (Table SVIII) with between 489 and 2023 peaks observed with S/N > 5 in the m/z range between 150 and 1000 g/mol. An assignment rate of 81–86% (including isotopologues) with a standard deviation ≤2% was achieved for all samples. The average number of formulae assigned per site had a low standard deviation (±11–18%) with the exception of the RMI samples (±46%) (Table SIX).

Altogether, 2385 unique monoisotopic molecular formulae were identified in the complete sample set (24% C_a_H_b_O_c_N_d_, and 76% C_a_H_b_O_c_). Woodland and grassland samples yielded the largest number of formulae (1675 and 1667, respectively), approximately double the number seen in the unburied samples (834). This is in stark contrast with the peatland samples that yielded numbers closer to those observed for the unburied tea, nevertheless, with distinct differences. For RMI samples, 29% more formulae (1079) were identified, while the trend was the opposite for the RMII samples, with 26% less formulae (620) identified. A closer inspection of the six RMI samples indicated that half of the samples had a larger average number of molecular formulae assigned (721), while the other samples had less than half (327) – an amount comparable to the RMII samples. The number of nitrogen compounds identified in the buried samples was in the order: grassland > woodland > RMI > RMII. This order follows the elemental analysis of the non-HWE buried samples (Table SII).

**Figure 4:**
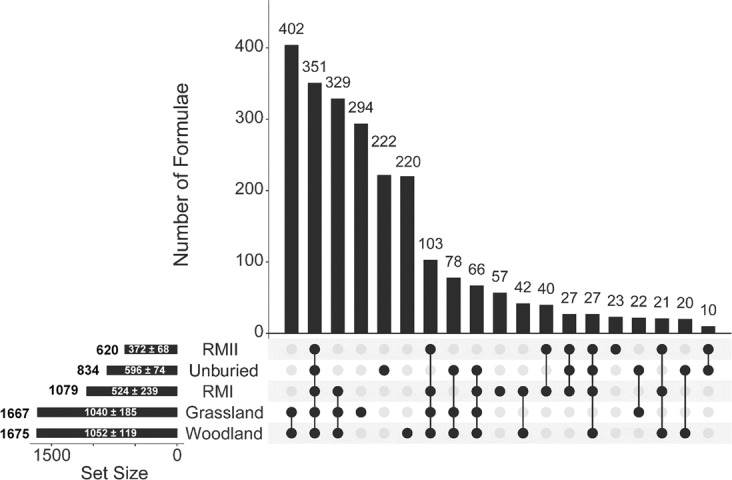
UpSet plot of the (-) ESI FT-ICR-MS data of the green tea samples. The plot shows for each site: the total number of unique total formulae (on the left); the average + standard deviation of formulae assigned (inside the horizontal bar). The vertical bars indicate the number of common assigned formulae for individual intersections defined by black circles and ordered in the descending population. Intersections I_1–3_ covered 46% of the assigned formulae, while the I_4–6_ and I_7–9_ intersection cumulatively extended the coverage to 77% and 88%, respectively.

The assigned molecular formulae were analysed with the help of an UpSet plot (Fig. 4, Table SX)^36^. These plots help visualize the distribution of compounds in a sample set, revealing a wealth of information. Molecular formulae for samples from *n* = 5 different environments can be represented by a maximum of 2^5^ − 1 = 31 intersections (*I*_1_ to *I*_31_) containing compounds common to *k* = 1–5 sample sets. Symbols $${I}_{i}(\begin{array}{c}5\\ k\end{array})$$ are used here to refer to individual intersections; these were ordered in the descending order of the number of formulae they contain. Formulae were only found up to $${I}_{28}(\begin{array}{c}5\\ k\end{array})$$ and the first 19 intersections shown in Fig. 4 represent more than 99% of the compounds.

A closer inspection of the intersection plots gives further insight into the molecular make-up of the tea buried in different sites. As *I*_1_ to *I*_9_ contained 88% of unique formulae, a detailed analysis presented here is limited to these intersections. The largest number of formulae (402) belongs to the $${I}_{1}(\begin{array}{c}5\\ 2\end{array})$$ group containing formulae found in woodland and grassland samples only. Similarity between the grassland and woodland samples is reinforced by the fact that the $${I}_{2}(\begin{array}{c}5\\ 5\end{array})$$ intersection, containing 351 formulae common to all sites, was the second most populated. It was interesting to see that the next most abundant intersection was $${I}_{3}(\begin{array}{c}5\\ 3\end{array})$$, containing 329 formulae from the grassland, woodland and RMI samples. In contrast, the $${I}_{22}(\begin{array}{c}5\\ 3\end{array})$$ intersection, containing formulae from the grassland, woodland and RMII, showed only 5 formulae (data not shown). This points towards similarities between the green tea buried at RMI and grassland/woodland sites and indicates differences between the green tea buried at the two RM sites. The *I*_4_ to *I*_6_ intersections are all of the $$(\begin{array}{c}5\\ 1\end{array})$$ type, i.e. contain unique formulae found in one site only. The 222 formulae identified in the unburied tea only, *I*_5_, represent compounds that were degraded in all environments. The numbers in the grassland (*I*_4_) and woodland (*I*_6_) intersections are significant (half of the *I*_1_ compounds), indicating the different effect these environments have on the tea samples. The next three intersections, *I*_7–9_, extend the coverage to 88% of compounds. The $${I}_{7}(\begin{array}{c}5\\ 4\end{array})$$ intersection, encompassing all but the unburied tea samples, showed 103 formulae. These represent compounds that were removed from samples in all environments. The next two intersections *I*_8_ and *I*_9_ are of $$(\begin{array}{c}5\\ 3\end{array})$$ and $$(\begin{array}{c}5\\ 4\end{array})$$ type, respectively. Neither includes the RMII site samples, both include unburied, woodland and grassland samples, and in the case of the $${I}_{9}(\begin{array}{c}5\\ 4\end{array})$$ also the RMI samples. These intersections therefore identify which compounds were removed from the RMI and RMII samples (78 formulae) and exclusively from the RMII samples (66 formulae), respectively. It is interesting to note that the $${I}_{24}(\begin{array}{c}5\\ 4\end{array})$$ intersection that contained formulae exclusively removed from the RMI samples had only three entries. These observations further emphasize the unique makeup of the tea buried in the RMII site. From the point of view of new formulae in the RM samples, these appear in $${I}_{10}(\begin{array}{c}5\\ 1\end{array})$$, $${I}_{15}(\begin{array}{c}5\\ 1\end{array})$$ and $${I}_{12}(\begin{array}{c}5\\ 2\end{array})$$ intersections, corresponding exclusively to RMI (57 formulae), RMII (23 formulae), and both RM samples (40 formulae), respectively. This is approximately 10 times less than the unique formulae detected in the woodland and grassland samples.

### Molecular level analysis of the FT-ICR-MS data

**Figure 5:**
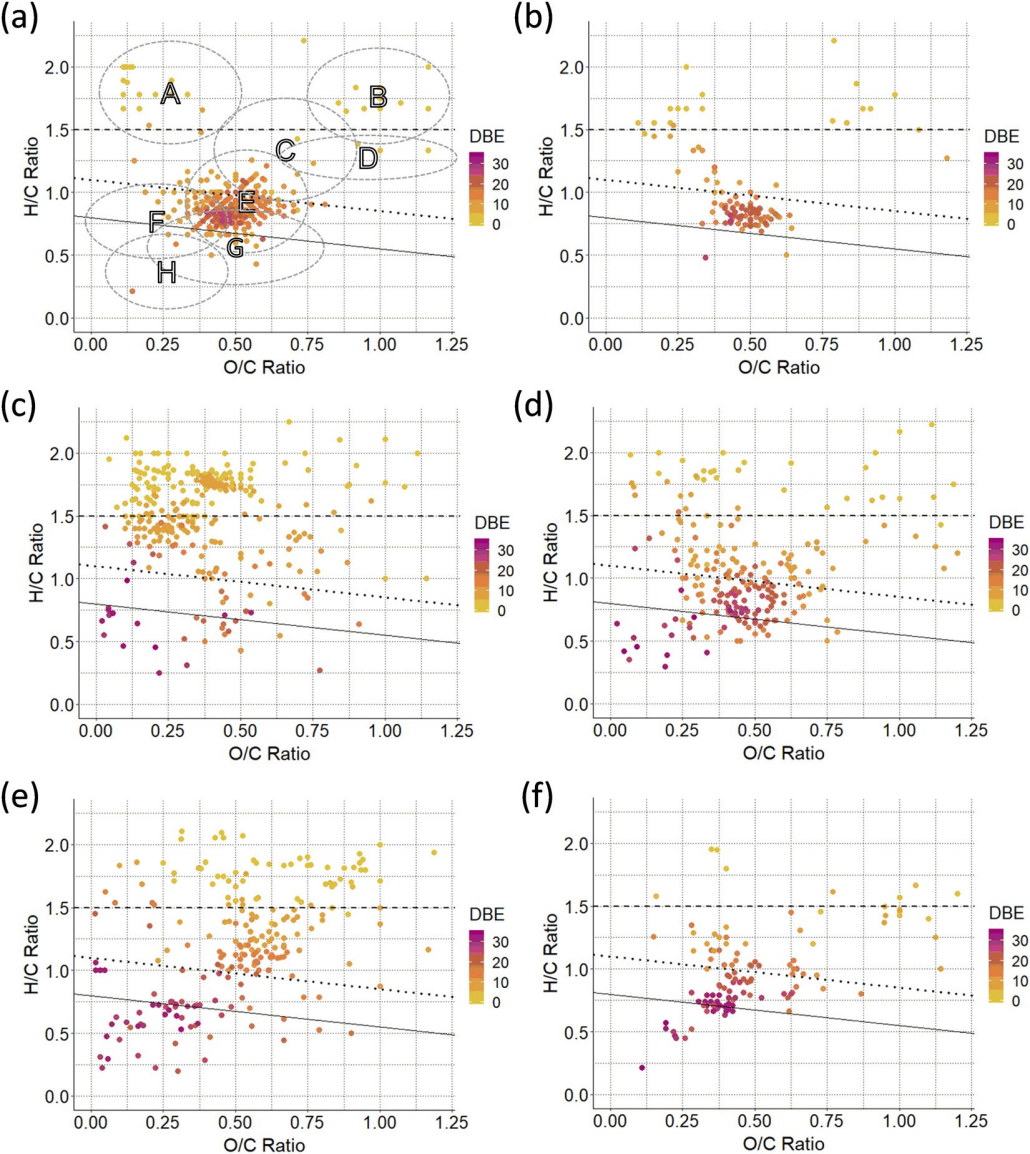
Van Krevelen diagrams for selected intersections identified by the UpSet plot. (**a**) preserved in all, I_2_; (**b**) produced in all I_7_; (**c**) produced in grassland only, I_4_; (**d**) produced in woodland only, I_6_; (**e**) removed in all environments, I_5_; (**f**) produced in RMI or RMII (I_10_ + I_12_ + I_15_). Colour coding denotes the DBE. The lines represent aliphatics (above the dashed line), aromatics (between the dotted and the full line) and condensed aromatics (below the full line) based on the AI_mod_ index^76^. Letters denote the following compound classes, A: fatty acids/lipids, B: carbohydrates, C: glycosides, D: nucleic acids, E: polyphenols, F: lignin- like compounds, G: oxidised polyphenols, H: condensed aromatics.

The H/C and O/C ratios were determined for the individual molecular formulae obtained from the FT-ICR-MS data and these were inspected using van Krevelen diagrams. These are presented for the representative samples in Supplementary Fig. S31. At the same time, the molecular formulae were examined by calculating their double bond equivalent (DBE) and a modified aromaticity index (AI_mod_)^37^. The results presented here focus on van Krevelen diagrams of selected intersections (Fig. 5) as identified by the UpSet plot; these intersections were also characterized by the oxygen class and mass distribution of their compounds (Supplementary Figs. S32 and S33).

#### Preserved in all environments (I_2_, 351 compounds)

Using the AI_mod_ index, the majority of these compounds (189, M_w_ 174–900 g/mol) can be classified as non-aromatic, followed by aromatic compounds (130, M_w_ 168–900 g/mol). Their mass distribution is centred between 200 and 600 g/mol, while their oxygen classes show a broad, skewed Gaussian distribution between O_2_ and O_17_ with a maximum at O_12_. The majority of molecular formulae identified in green tea by others are present in this intersection^22,38–40^. These include fatty acids, namely, oleic acid, palmitic acid, linolenic acid, linoleic acid, stearic acid, myristic acid^38,39^, while others are tentatively assigned to pentadecanoic acid and palmitoleic acid. The glycoside region contains molecules, such as rutin and kaempferol glycosides^22,41^. The molecular formula C_12_H_22_O_11_ represents many disaccharides, including sucrose and trehalose that were identified by NMR. All major catechins were identified, the most abundant being ECGC, however dimers and oxidative products, such as theaflavins, were also found. Removal of catechins in some samples, as detected by NMR, was therefore not complete. There is very little of condensed aromatics, all of which are low molecular weight compounds (12, M_w_ 162–302 g/mol).

#### Produced in all environments (I_7_, 103 compounds)

Only 103 (61 non-aromatic, 39 aromatic, 3 condensed aromatic) new compounds were detected in all soil environments. Their mass distribution was bimodal with maxima at 300 and 600 g/mol, respectively, while their oxygen class distribution peaked at O_15_. The non-aromatic molecules were a mix of fatty acids and glycosides with a few carbohydrates. Molecular formulae of theaflavins and theanapthoquinones^42,43^, oxidative products of the green tea typically found in the black tea, were identified.

#### Removed from all environments (I_5_, 222 compounds)

The majority of these compounds are non-aromatic (166, M_w_ 150–800 g/mol) falling into the glycoside area, followed by fatty acids, carbohydrates and condensed aromatics. Their mass distribution was Gaussian, centred at around 600 g/mol, showing a fairly even range of masses, while higher oxygen classes were more populated. Few tentative assignments were made, namely C_5_H_10_O_5_ which is likely arabinose and C_15_H_18_O_9_, which has been identified as caffeoyl-d-glucose. Many of the *I*_5_ molecular formulae are related to *I*_2_ formulae by a specific molecular unit such as oxygen, or isoprene units. Comparing the produced in all and removed from all sites, more glycosides were removed than were produced.

#### Produced only in grassland (I_4_, 294), only in woodland (I_6_, 220), and only in peatland (I_10_ + I_12_ + I_15_, 120 compounds)

The van Krevelen plots show a clear difference between molecules produced in grassland only (*I*_4_) and woodland only (*I*_6_), with grassland samples having more fatty acid compounds, while woodland samples contain more flavonoids/glycosides. In addition, more aromatic molecules were produced in the woodland (57 aromatic, 27 condensed) compared to the grassland (17 aromatic, 12 condensed) samples. The molecular weight distribution in the grassland samples shows mainly low M_w_ compounds (centred on 400 g/mol), while the woodland produced larger molecules (centred on 600 g/mol). The grassland compounds belong to low oxygen classes (centred on O_6_), while the woodland compounds have a broad distribution up to O_20_. A few amino acids (histidine and tryptophan) could be identified in the grassland samples only, while xanthines such as theophylline as well as certain theaflavin mono/digallates were found only in the woodland samples. The abundance of fatty acids in the grassland samples (also seen by NMR) is a striking feature.

Compared to the grassland and woodland, far fewer compounds were produced in the peatland, over 70% of which were found in the oxidised polyphenols and glycoside regions of the van Krevelen with a bimodal distribution in the oxygen classes centred on O_7_ and O_16_. A number of their formulae belong to large compounds, e.g. theasinensin digallate and its homologues.

The stress induced molecule, mannitol, was not found in these intersections; it only appeared in the *I*_3_ intersection, in agreement with NMR data.

### Chemometric analysis of the FT-ICR-MS spectra of the HWE green tea samples

**Figure 6:**
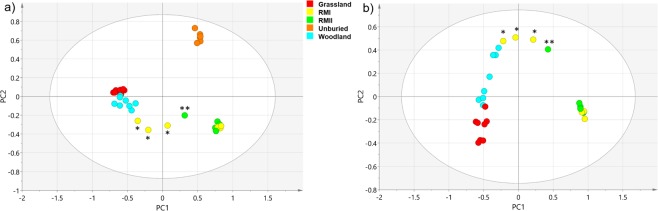
PCA score plot from the (-) ESI FT-ICR-MS data. Each circle represents an individual HWE tea sample mass spectrum. (**a**) analysis of unburied tea, tea buried in grassland, woodland, RMI and RMII; (**b**) analysis of only buried tea samples. The legend indicates the colour coding for each sample site. The Hotelling’s T^2^ ellipse represents 95% confidence interval. Outlying RMI and RMII samples are marked with a single and double asterisks, respectively.

The PCA analysis of the FT-ICR-MS spectra using two principle components accounted for 68% of the total variance (53 and 16% by PC1 and PC2, respectively). The percentage of the total predicted variance was 62% (47 and 15% by PC1 and PC2, respectively). The MS PCA score plots (Fig. 6) indicate significant between-group variation with a tight, well-separated cluster of the unburied tea (two samples measured in triplicate confirming the reproducibility of the method).

Grassland and woodland samples appeared close together in one area of the score plot, with more variation in the woodland sample set. Three RMI samples were located closer to the woodland samples, while the rest overlaid with the RMII samples. Only one RMII sample trended towards the RMI/woodland samples, while the rest clustered tightly in a separate area of the score plot.

The PCA analysis of the FT-ICR-MS spectra excluding the unburied tea samples using two principle components accounted for 72% of the total variance (60 and 12% by PC1 and PC2, respectively). The percentage of the total predicted variance was 65% (56 and 10% by PC1 and PC2, respectively). Removing the unburied tea samples from the analysis (Fig. 6b) preserved the features discussed above and accentuated within-group differences in the grassland and woodland samples, as seen by their spread along the PC2. This was not the case for the RM samples, which did not change their clustering pattern. This PCA score plot shows a significant “arch effect”, which may occur with multivariate methods where the data is projected onto low dimensions. This may reflect a single dominant gradient in the data and that the samples are highly structured according to PC1. Nevertheless, as discussed^44^ the arch effect is not necessary artefactual. Since the MS data show a similar ordination of samples as the NMR data, this is also likely the case here.

PCA loadings for the analysis with and without the unburied tea are shown in Supplementary Figs. S34–S36, respectively. The majority of the discriminating molecular formulae were assigned to the most likely compounds, considering those previously found in green tea^18–20,22,30,40,42,45,46^.

Based on well-separated PCA scores, PLS-DA was performed next. Similar to the analysis of NMR data, the PLS-DA of the MS data accentuated the within-group differences in the grassland and woodland samples, while it did not change the clustering pattern of the RM samples (Supplementary Fig. S37). The corresponding HCA dendrogram (Supplementary Fig. S38) mirrors the clustering of the PLS-DA score plots. The overlaid biplots singled out the molecular formulae/compounds that contributed most to the PLS-DA score plot definition. All were already identified as significant by the PCA and are highlighted in the figure legends of Supplementary Figs. S35 and S36.

The biplots (Supplementary Fig. S37) reflect higher concentrations of EGCG, ECG/CG, gallic acid and C_24_H_20_O_15_ in the RM samples, while trehalose was particularly elevated in the grassland samples. EGC/GC, C_16_H_14_O_9_, flavonols (such as myricetin), C_30_H_26_O_13_ (catechin dimers), epicatechin trimer procyanidin C1, as well as, polyphenol flavonoids (such as theasinensin B and C) were elevated in woodland and some RMI samples.

## Discussion

Both high resolution NMR spectroscopy and MS provided a wealth of information regarding changes to the composition of the HWE buried green tea. Analysis of the identified molecular formulae yielded insights into the compound class distribution in individual intersections as defined by the UpSet plot analysis together with their attributes such as molecular mass, aromaticity index and oxygen class. Furthermore, the simplicity of the green tea has allowed the assignment of several individual molecules from the MS and NMR data. Such level of detail has not been achieved to date for buried plant litter or labile SOM due to the complexity of these mixtures, limiting their analysis to compound classes only^47–49^.

Treated chemometrically, both NMR and MS data of the HWE buried green tea revealed significant differences between samples. As PCA showed inter-group variations above the level of intra-group differences, a supervised dimensionality reduction method, PLS-DA, was employed to classify the samples. Reflecting unique transformations of the green tea buried in different soils, this approach has successfully provided molecular signatures of green tea decomposition, including compounds responsible for the ordering of the samples. It is remarkable that despite the several orders of magnitude difference in sensitivity between NMR and FT-ICR-MS, both techniques classified samples in a very similar way. Not only were the woodland and grassland samples clearly separated from the peatland samples, but also some damaged peatland samples (RMI) separated from the samples from the peatland site under restoration (RMII). Labile SOM is sensitive to land-use changes or management^50–52^ and therefore analysis of the HWE buried tea has the potential to report on peatland ecosystem health. These observations will form basis of a more extensive study focusing on peatlands (raised and blanket bogs) in different condition.

### Relating the molecular changes to biotic and abiotic factors

What changes are expected to occur upon burial of tea bags and what drives them? Lipton tea bags are made from a non-woven material that will stop macrofauna from getting inside^8^. Therefore, only physical, microbial and chemical degradation of the buried tea is possible. The observed molecular transformations are the result of a combination of processes which resulted in the (i) disappearance or reduction in the amount of compounds, either by their biodegradation, chemical modification or physical removal via dissolution in water and water flux; (ii) appearance of new, smaller molecules as a consequence of the above processes; (iii) partial degradation of green tea polymers that are not soluble, producing smaller, water extractable poly/oligomers; (iv) oligo/polymerization of compounds; and finally (v) the appearance of small or large molecules of biological origin that are either excreted by, or extracted from, microorganisms after they have been imbedded in the tea samples.

Temperature is one of the major factors affecting decomposition of litter and SOM^53,54^; burial of tea in winter months with relatively stable but low temperature will slow down biotic decomposition^6^, allowing any differences due to other factors to be accentuated. Stable rainfall/snowfall at the sites of the buried tea during winter meant that moisture was not the limiting factor across soils in same region^55^. Another abiotic factor, pH, is an important factor that affects the enzymatic and chemical activity and therefore the level of SOM transformation^56–58^. Access to oxygen is yet another inherent feature that, e.g. in waterlogged peatlands, is a limiting factor for some enzymatic activities^56,59^.

Soil exoenzymes produced by microorganisms are proximate agents of OM decomposition^58^ and can largely be classed as hydrolytic (breakdown of labile compounds, e.g. carbohydrates) and oxidative (responsible for transformation of less labile compounds, e.g. aromatics)^58,60,61^ Hydrolytic enzyme activities are dependent on labile carbon content, temperature, soil C/N ratio and pH, while oxidative enzymes are limited mainly by pH and the availability of oxygen^58,62^. While the C/N ratio was not measured for the soils examined in this study, it was for the unburied and buried tea, which can be compared to studies examining the C/N ratio of buried litter. The C/N ratio decreased after burial, which is in an agreement with the incubation studies of litter that reported a decrease in C/N believed to be associated with either the increase in microbial N-containing metabolites or non-decomposable N-containing compounds^15^. Sinsabaugh *et al*. examined the activity of the seven-most widely measured soil enzymes, using data from 40 ecosystems, and found that the oxidase enzyme activity and decomposition increased with pH, while hydrolase enzyme activity showed a weak or strong negative correlation with pH^58^. For the soil types in this study, the C/N ratio of the buried tea decreased in order of peatland > woodland > grassland, while the pH of the soils decreased in the order of grassland > woodland > peatland. Thus, both factors suggest that biotic decomposition, and specifically oxidative enzyme activity, was higher in grassland soils. This is supported by the fact that the greatest mass loss and greatest number of compounds produced (microbial N-containing plus tea decomposition products) was for tea bags buried in the grassland. The decrease in decomposition from grassland to peatland matches exactly with the results of the initial Tea Bag Index study^8^.

### Molecular level transformations

The molecular transformations that occur when litter decomposes have been summarised generally as a decrease in carbohydrates and increase in aromatics and aliphatics^47,63^, through hydrolysis or oxidative processes. As tea is a simpler mixture compared to both plant litter and SOM, decomposition can be studied to the highest level of specific molecular changes. For example, considering that black tea is produced by oxidation of the same tea leaves as green tea (*Camellia sinensis*), the major products of which are oxidized catechins such as thearubins, it is possible that oxidative processes in soils could produce the same compounds that are found in black tea^42,64^. Inspection of HWE buried green tea using high resolution MS provided evidence for this. Molecules such as catechin dimers or trimers, as well as thearubins such as theasinensin B and C, were found in woodland and peat samples. However, other molecules such as theaflavin-3-gallate, associated with black tea^42^, were found in all samples, including the unburied tea. It is possible that these compounds were already present in unburied tea at very low concentrations and were detected due to the high sensitivity of FT-ICR-MS.

Appearance of cold stress molecules, trehalose and mannitol, in the grassland, woodland and some RM samples is intriguing and has not yet been reported for buried litter. Trehalose is excreted by fungi and its release accompanies root nodule senescence^65^. Together with mannitol, trehalose is known to protect cells from frost damage^35,65,66^. The appearance of these molecules is likely a consequence of the winter burial period; additional experiments performed in summer/autumn are required to prove this hypothesis.

MS analysis also uncovered an increased presence of lipids in the grassland samples. While their exact structures could not be determined, the majority form homologous series differing by the number of oxygens or CH_2_ groups. The abundance of unique hydroxyl and methyl branched fatty acids in grassland compared to woodland soils in colder months has been reported previously due to the greater presence of gram negative bacteria or actinobacteria than gram positive bacteria^67,68^.

Partially hydrolysed arabinogalactans were detected in both the damaged peatland (RMI) and the peatland undergoing restoration (RMII) (but not grassland and woodland samples). Large insoluble arabinogalactans, which have been reported in green tea^20^, are unobservable by liquid state NMR, therefore these must have been produced by hydrolysis which could have occurred abiotically (due the acidic pH of peatlands) and/or biotically (by peat-inhabiting hydrolytic planctomycetes that are active even at low temperatures)^69,70^.

Major differences were observed in aromatic regions of the NMR spectra. Catechins – phenolic molecules – were mostly degraded in the RMI samples (the damaged peatland site). This finding can be explained by the enzyme latch theory, which states that phenolics are elevated in a well-functioning peatland with persistent anoxia and low pH, which hinder the activity of oxidative enzymes like phenol oxidase^56^. Functioning peatlands have an anoxic zone below the predominant water table. Following the instructions of the TBI project, the samples were buried at a depth of 8 cm. This is above the typical water table in both RMI and RMII sites (~15 cm). However, it is possible that during the wet winter months (snow also laid on these sites for two months of the burial period), these could have been submerged under water and hence starved from oxygen. It is also possible that winter temperatures limited the enzymatic activity^71^. One cannot rule out that different microbial communities were present in the damaged and restored sites at the start of the study, which could have led to this difference. Concomitant examination of the microbial community structure and enzyme activity is therefore needed to clarify these observations.

## Conclusions

Untargeted analysis using high resolution NMR and MS techniques was performed to characterise the transformations of hot water extracts of green tea buried in grassland, woodland and peatlands as a proxy to labile SOM. Our results demonstrate that the molecular composition of green tea changes upon burial in a soil type specific manner. The abiotic and biotic factors that caused these changes were discussed. NMR and MS reported on major and minor products of the biochemical transformations of the buried tea, respectively. Furthermore, the use of a simpler mixture allowed the identification of specific molecules that represent the differences in decomposition in individual soil types. Differences were seen in the HWE tea buried between peatland sites that were damaged and ‘under restoration’, which shows that the molecular characterisation of buried tea could be used to examine the effect of land-use or management changes. The data from this study, together with their chemometric interpretation, provide a basis on which to extend our knowledge of soil decomposition and C-cycling through a widely used litter material – green tea – as a proxy to the transformation of labile SOM.

## Methods

### Tea bag burial sites

Four sites were investigated in this study (Supplementary Fig. S1). The first two were on a raised peatland (called Red Moss of Balerno) located in Balerno outside of Edinburgh, Scotland (55°51′38.4″N 3°20′15.4″W). This is a designated Site of Specific Scientific Interest as one of the remaining raised bogs in Scotland. The bog has undergone historic damage from grazing, burning and drainage. In the last 30 years its western part has undergone restoration in the form of drain blocking using dams. Two sites were examined, one nearest to the dams (RMII, 55°51′37.7″N 3°20′17.4″W) and a site on the NE side, which is currently unrestored (RMI, 55°51′43.6″N 3°20′04.1″W). The third was a woodland located 100 m to the west of the Red Moss peatland near Threipmuir reservoir (55°51′37.0″N 3°19′59.9″W). The soil type according to the Soil map of Scotland is a mineral gley^72^. The forth site was a species rich grassland (55°52′23.3″N 3°12′11.8″W) located on the south side of the Pentland hills; samples were placed on the edge of arable plots. The soil type according to the Soil map of Scotland is brown soil^72^. The average pH of each soil is given in Table SI.

### Tea bag burial and extraction

Eleven sets of non-woven rooibos (Lipton, EAN 87 22700 18843 8) and green tea (Lipton, EAN 87 22700 05552 5) bags were weighed (average weight 1.98 g) and buried at the two peatland sites and the woodland site, while 12 sets were buried at the grassland site in November 2017. Winter burial was chosen to prevent influence of high temperatures and root penetration on the tea decomposition. In addition, the winter months generally provide easier access to the sites, considering conservation activities and staff availability. One rooibos tea bag and one green tea bag were buried 15 cm apart, both at a depth of 8 cm. Three sets of bags were buried 2 m apart and the rest diagonally outwards. Four sets of tea bags were buried 15 m from the centre and four sets 30 m. The samples were collected after 96 days. Out of the 90 tea bags buried, 82 were recovered. To enable submission of data to the Tea Bag Index project, 4–6 bags were taken from each set and processed according to the Tea Bag Index protocol^8^.

The remaining tea bags were dried and weighed with any soil particles on the outside removed. The tea taken out of each bag and weighed, including string plus label, separately before being balled milled. The average tea bag weight loss per site are provided in Table SI.

Samples for NMR and MS analysis were prepared by HWE using the following protocol: 150 mg of ground tea was heated to 80 °C in Milli-Q water for 30 minutes using a microwave. The supernatants were filtered using glass fibre filters (GF/C, Whatman) and freeze dried. On average 15 mg of the freeze dried green tea extract was obtained per tea bag.

### Elemental analysis

The elemental composition (C, H, N) of ground tea samples was determined using an ThermoScientific FlashSmart analyser. C, H, N was determined for selected tea bags with duplicates from each burial site. Elemental analysis of tea bags that were unburied but HWE and unburied and not HWE was also conducted (Table SII).

### ^1^H NMR spectroscopy

For all NMR analysis of unburied and buried green tea samples (Bruker 600/800 MHz AVANCE III with 5 mm TCI cryoprobes), 5 mg of freeze dried HWE tea was dissolved in 600 µL of D_2_O (100% deuterated, Sigma Aldrich). A 1D NOESY based water suppression pulse sequence was used to acquire the 1D ^1^H spectra. The following parameters were used: 64 scans, 9 s relaxation delay, 1.3 s acquisition time, 40 ppm spectral width and 80% pulsed field gradients during a 100 ms the mixing time. Overall experimental time was 12 minutes per sample. Line broadening of 1 Hz and zero filling were applied prior to Fourier transformation. Altogether 34 spectra were acquired including samples of the unburied tea (3), and samples buried in woodland (8), grassland (9), the damaged peatland (RMI, 8) and the peatland under restoration (RMII, 6).

### Parameters of additional NMR experiments

*T*_1_ relaxation experiments were performed on representative green tea samples using a modified inversion recovery pulse sequence on the 800 MHz spectrometer equipped with a TCI cryoprobe at 300 K. The following parameters were used: inversion recovery times of *τ*_*min*_ = 1 ms and *τ*_*max*_ = 1400 ms and 16 relaxation points, 4 dummy scans, 128 scans, 40 ppm spectral width, 2 s relaxation delay and 1 s acquisition time. Water presaturation was applied during the prescan relaxation delay and relaxation intervals.

*T*_2_ experiments were acquired on the same sample set using a CPMG sequence on the 800 MHz spectrometer. The parameters used were identical to those used for the *T*_1_ experiment apart from the following: relaxation intervals: *τ*_*min*_* = *20 ms, *τ*_*max*_ = 564 ms, number of relaxation points = 12.

2D DOSY spectra were acquired using the convection compensated pulse sequence (Bruker pulse program ledbpgp2s) at 300 K using the 800 MHz NMR spectrometer. The gradient strength of the pulse field gradients was increased from 5% to 95% of a set maximum of 53 G/cm over 16 points. The diffusion time was set to 100 ms. The following additional parameters were used: 2 dummy scans, 256 scans, 2 s relaxation delay, 40 ppm sweep width and 2 s acquisition time.

1D CSSF TOCSY^73^ spectra were acquired using the Bruker pulse program selcssfdizs at 300 K using the 800 MHz NMR spectrometer. The carrier was positioned on a chemical shift of the protons of interest (H-1b/6b of mannitol, H-2 trehalose and H-1 amylopectin). The CSSF was set to eliminate signals at a distance of 12.5 Hz. A 40 ms Gaussian pulse was used. 12 increments were acquired with 2 scans each. A mixing time of 100 ms and 120 ms was used for mannitol/trehalose and amylopectin, respectively.

2D ^1^H, ^13^C HSQC spectra were acquired using the Bruker pulse program hsqcedetgpsisp2.3 and the following parameters: 2048 and 2048 complex points in *t*_2_ and *t*_1_, respectively, spectral widths of 10 and 150 ppm in *F*_2_ and *F*_1_, yielding *t*_2_ and *t*_1_ acquisition times of 128 and 34 ms, respectively. Sixteen scans were acquired for each *t*_1_ increment using a relaxation time of 1.5 s. The polarisation transfer was optimised for ^1^*J*_CH_ = 150 Hz. Forward linear prediction to 4096 points was applied in *F*_1_. A zero filling to 4096 was applied in *F*_2_. A cosine squared window function was used for apodization prior to Fourier transformation in both dimensions. The overall acquisition time was 15 hours per spectrum.

### FT-ICR-MS

1 mg samples of HWE freeze dried tea extracts were prepared in 50:50 MeOH/H_2_O and diluted to a final concentration of 0.1 mg/ml. Analysis was performed on a 12 T SolariX (Bruker Daltonics) with an Infinity cell. Negative mode Electrospray Ionization was used and the sample was sprayed at a rate of 200 µl/hour. Ion accumulation time and time of flight, were set to 0.2 s and 0.6 ms, respectively. 200 transients at 4 MW were summed. Spectra of 3 unburied (in triplicate) and 28 buried green tea samples were collected and calibrated using the formulae of compounds present in green tea^22,40^. A signal to noise ratio > 5 and an error threshold of ±0.5 ppm were used to assign molecular formulae in Formularity^74^ with the following elemental constraints ^12^C0–80 ^1^H0–160 ^16^O0–40 ^14^N0–8.

### Chemometric analysis of 1D 1H NMR and FT-ICR-MS data

The chemometric analysis (PCA, PLS-DA, HCA) was performed using SIMCA 14.1. A ^1^H NMR spectral region of 0.5–10 ppm, excluding the interval between 4.65–4.75 ppm that contains the residual HOD signal, was used. The bin width was 0.01 ppm and regional spectral alignment prior to integration was done using home written scripts based on icoshift^75^. Individual masses were used for the analysis of the MS data with sum total normalisation applied to each sample. Pareto scaling was used for both NMR and MS data.

## Supplementary information

Supplementary Information 

## Data Availability

The datasets generated during and analysed during the current study are available from the corresponding author.
