# Supplementary material for: Molecular level study of hot water extracted green tea buried in soils - a proxy for labile soil organic matter
Source: Sci Rep. 2020 Jan 30;10:1484. doi: 10.1038/s41598-020-58325-8 (PMC6992787; doi:10.1038/s41598-020-58325-8)
Supplement: Supplementary file 1 — Supplementary Information [file 41598_2020_58325_MOESM1_ESM.pdf]

Supplementary Information

Molecular level study of hot water extracted green tea buried in soils - a proxy for labile soil organic matter

Nicholle G. A. Bell*, Alan J. Smith, Yufan Zhu, William H. Beishuizen, Kangwei Chen, Dan Forster, Yiran Ji, Elizabeth A. Knox

The Supplementary Information contains the following:

Section S1: Experimental details

Figure S1. Locations of the tea bag burial sites.

Table SI. Average pH of soil and average mass loss of buried tea bags.

Table SII. C, H, N elemental analysis of representative ball-milled green tea samples.

Section S2. Assignment of specific NMR resonances from buried and unburied green tea spectra

Figure S2. 800 MHz ^{1}H spectrum of the HWE unburied tea with assignment of resonances.

Figure S3. Partial 800 MHz 2D ^{1}H, ^{13}C HSQC NMR spectra of HWE unburied tea highlighting the signals of amylopectin.

Figure S4. Catechin region of the 800 MHz 2D ^{1}H, ^{13}C HSQC NMR spectra of HWE green tea from individual sites.

Figure S5. Anomeric regions of the 800 MHz 2D ^{1}H, ^{13}C HSQC NMR spectra of HWE green tea from individual sites.

Figure S6. Carbohydrate region of the 800 MHz 2D ^{1}H, ^{13}C HSQC NMR spectra of HWE green tea from individual sites.

Figure S7. 800 MHz 1D CSSF-TOCSY spectra of mannitol and trehalose of the HWE of tea buried in grassland.

Section S3: ^{1}H NMR spectra of buried and unburied green tea extracts used for PCA

Figure S8: 600 MHz ^{1}H NMR spectra of HWE green tea buried at the woodland site.

Figure S9. 600 MHz ^{1}H NMR spectra of HWE green tea buried at the grassland site.

Figure S10: 600 MHz ^{1}H NMR spectra of HWE green tea buried at the damaged peat bog site (RMI).

Figure S11: 600 MHz ^{1}H NMR spectra of HWE green tea buried at the peat bog site under restoration (RMII).

Section S4. Analysis of ^{1}H relaxation times of the HWE green tea samples

Table SIII. T_1 relaxation times of protons resonating in the three spectral regions.

Table SIV. Fast T_2 component of the ^{1}H relaxation times.

Table SV. Slow T_2 component of the ^{1}H relaxation times.

Figure S12. 800 MHz 2D T_1 relaxation map of the HWE unburied green tea sample.

Figure S13. 800 MHz 2D T_1 relaxation map of the HWE green tea sample buried in RMI.

Figure S14. 800 MHz 2D T_1 relaxation map of the HWE green tea sample buried in RMII.

Figure S15. 800 MHz 2D T_1 relaxation map of the HWE green tea buried in woodland.

Figure S16. 800 MHz 2D T_1 relaxation map of the HWE green tea buried in grassland.

Figure S17. 800 MHz 2D T_2 relaxation map of the HWE unburied green tea sample.

Figure S18. 800 MHz 2D T_2 relaxation map of the HWE green tea buried in RMI.

Figure S19. 800 MHz 2D T_2 relaxation map of the HWE green tea buried in RMII.

Figure S20. 800 MHz 2D T_2 relaxation map of the HWE green tea buried in woodland.

Figure S21. 800 MHz 2D T_2 relaxation map of the HWE green tea buried in grassland.

Section S5. Analysis of DOSY spectra of the HWE green tea samples

Figure S22. The F_1 projection of 800 MHz 2D DOSY spectra of HWE green tea samples.

Figure S23. DOSY Analysis of the HWE unburied green tea.

Figure S24: DOSY analysis of the HWE green tea buried in the RMI site.

Figure S25: DOSY analysis of the HWE green tea buried in the RMII site.

Figure S26: DOSY analysis of the HWE green tea buried in the woodland site.

Figure S27. DOSY analysis of the HWE green tea buried in the grassland site.

Section S6. PCA of ^{1}H NMR spectra of buried and unburied HWE green tea

Figure S28: PCA loadings plots associated with the score plot shown in Fig. 6.

Figure S29. PLS-DA score plot based on the 1D ^{1}H 600 MHz NMR spectra of the HWE buried green tea samples without the unburied green tea.

Figure S30. HCA dendrogram based on the PLS-DA of 1D ^{1}H 600 MHz NMR spectra of the HWE buried green tea samples without the unburied green tea.

Section 7. FT-ICR-MS analysis of buried and unburied HWE green tea

Table SVIII. Statistics for the FT-ICR-MS spectra of green tea samples.

Table SIX. Average assignment statistics for the FT-ICR-MS spectra of green tea samples.

Table SX. Summary of the number of compounds and % relative to unburied tea for each intercept from the UpSet plot given in Fig. 4.

Figure S31. Van Krevelen plots for representative samples whose NMR spectra are shown in Fig. 1.

Figure S32. Oxygen series for selected UpSet intersections.

Figure S33. Mass count for selected UpSet intersections.

Section 8. PCA of FT-ICR-MS spectra of unburied and buried HWE green tea

Figure S34. PC2 loadings plot from the PCA of FT-ICR-MS spectra with unburied green tea.

Figure S35. PC1 loadings plot from the FT-ICR-MS PCA of the MS spectra without unburied green tea.

Figure S36. PC2 loadings plot from the FT-ICR-MS PCA of the MS spectra without unburied green tea.

Figure. S37. PLS-DA score plot based on the MS spectra of the HWE buried tea samples without unburied green tea.

Figure S38. HCA dendrogram based on the PLS-DA of MS spectra of the HWE buried tea samples without unburied green tea.

References

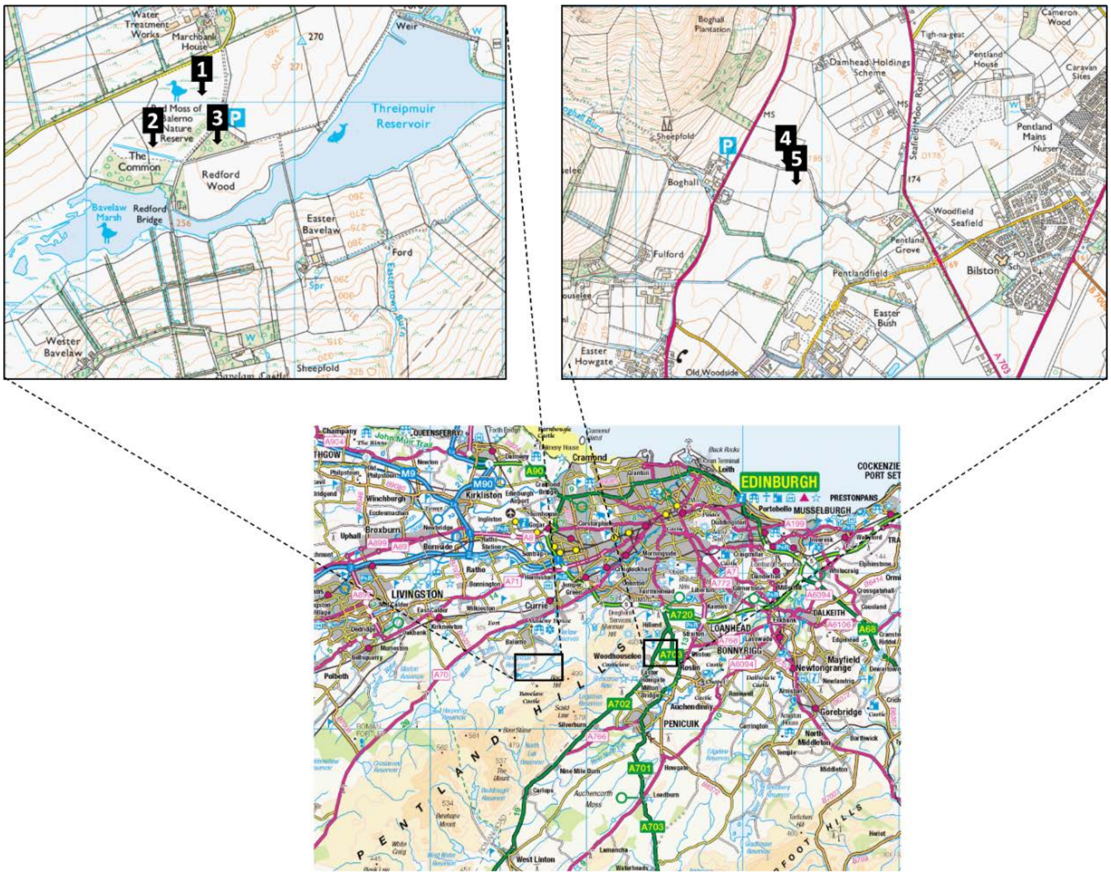

Fig. S1. Locations of the tea bag burial sites. 1: RMI, 2: RMII, 3: woodland, 4 and 5 species rich grassland. Map created in Edina Digimap.

Table SI. Average pH of soil and average mass loss of buried tea bags

Burial Site	Average mass loss of green tea after burial/ g [relative standard deviation/ ±%]	Average soil sample pH [relative standard deviation/ ±%]
RMI	0.90 [±12]	4.4 [± 0.4]
RMII	0.83 [± 7]	4.1 [± 0.4]
Woodland	1.05 [± 9]	5.1 [± 0.5]
Grassland	1.07 [± 5]	6.4 [± 0.3]

Table SII. C, H, N elemental analysis of representative ball-milled green tea samples

Sample	N / %	C / %	C/N	H / %
G37_Grassland	7.08	51.66	7.30	6.62
G58_Woodland	5.85	52.55	8.98	6.59
G27_RMI	5.73	51.87	9.05	6.96
G43_RMII	5.4	50.68	9.39	6.81
G12_Unburied	3.92	47.73	12.17	6.10
G12_Unburied_HWE*	2.33	45.15	19.38	5.28

* G12_Unburied_HWE: elemental analysis was performed on the hot water extract.

Section S2. Assignment of specific NMR resonances from buried and unburied green tea spectra

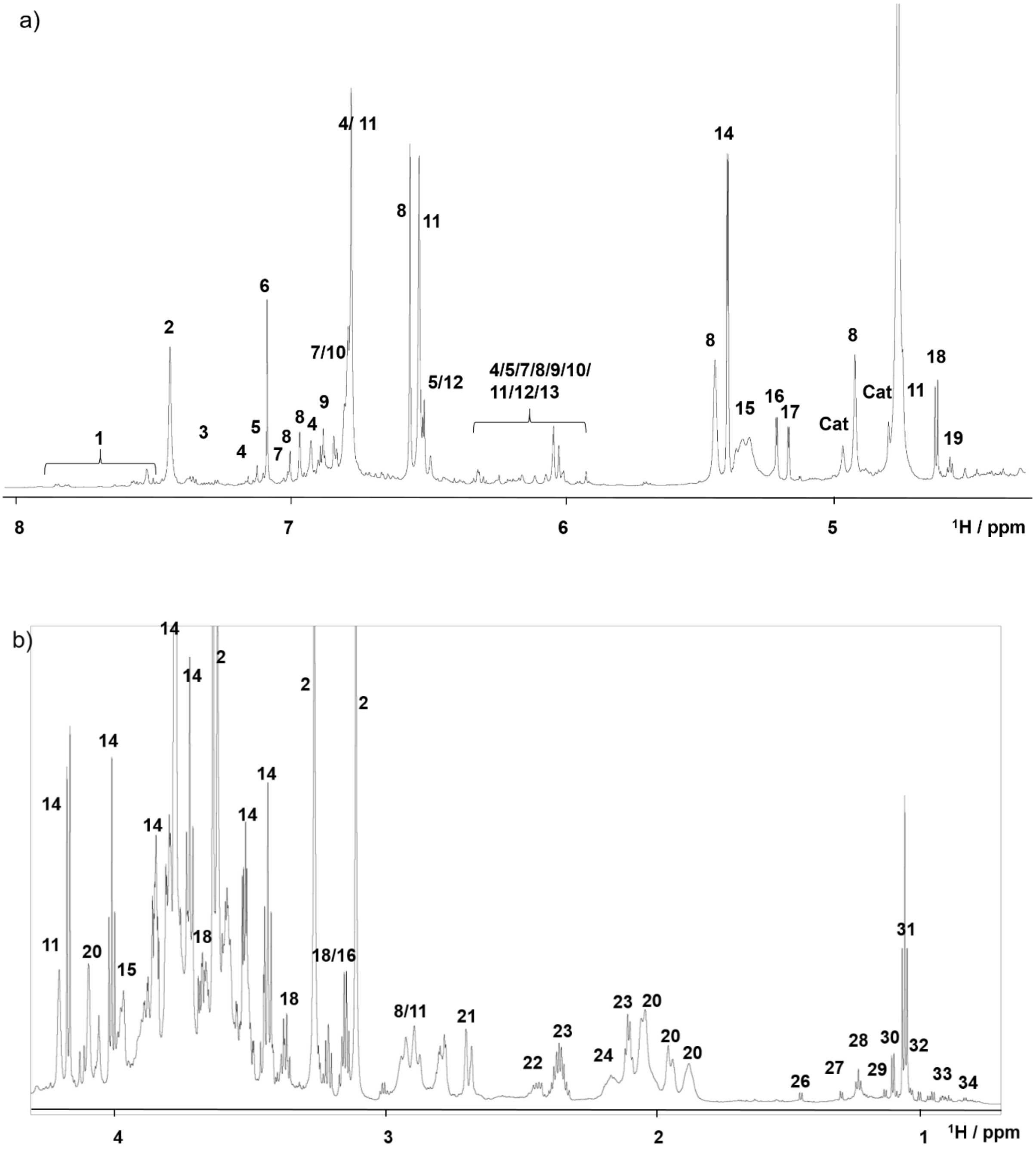

Fig. S2. 800 MHz ^{1}H spectrum of the HWE unburied tea with assignment of resonances. **1.** theobromine/chlorogenic acid; **2.** caffeine; **3.** p-coumaroyl quinic acid; **4.** CG; **5.** GCG; **6.** gallic acid; **7.** EC; **8.** EGC; **9.** CG; **10.** ECG; **11.** EGCG; **12.** GC; **13.** flavonoids; **14.** sucrose; **15.** amylopectin; **16.** α-glucose; **17.** 2-O-(β-L-arabinopyranosyl) myo-inositol; **18.** β-glucose; **19.** unknown, **20.** quinic acid; **21.** aspartic acid; **22.** Glutamic acid; **23.** glutamine; **24.** unknown; **25.** alanine; **26.** threonine; **27.** fatty acids; **28.** unknown; **29.** unknown; **30.** theanine; **31.** valine; **32.** isoleucine; **33.** leucine. Cat. Denotes multiple catechins.

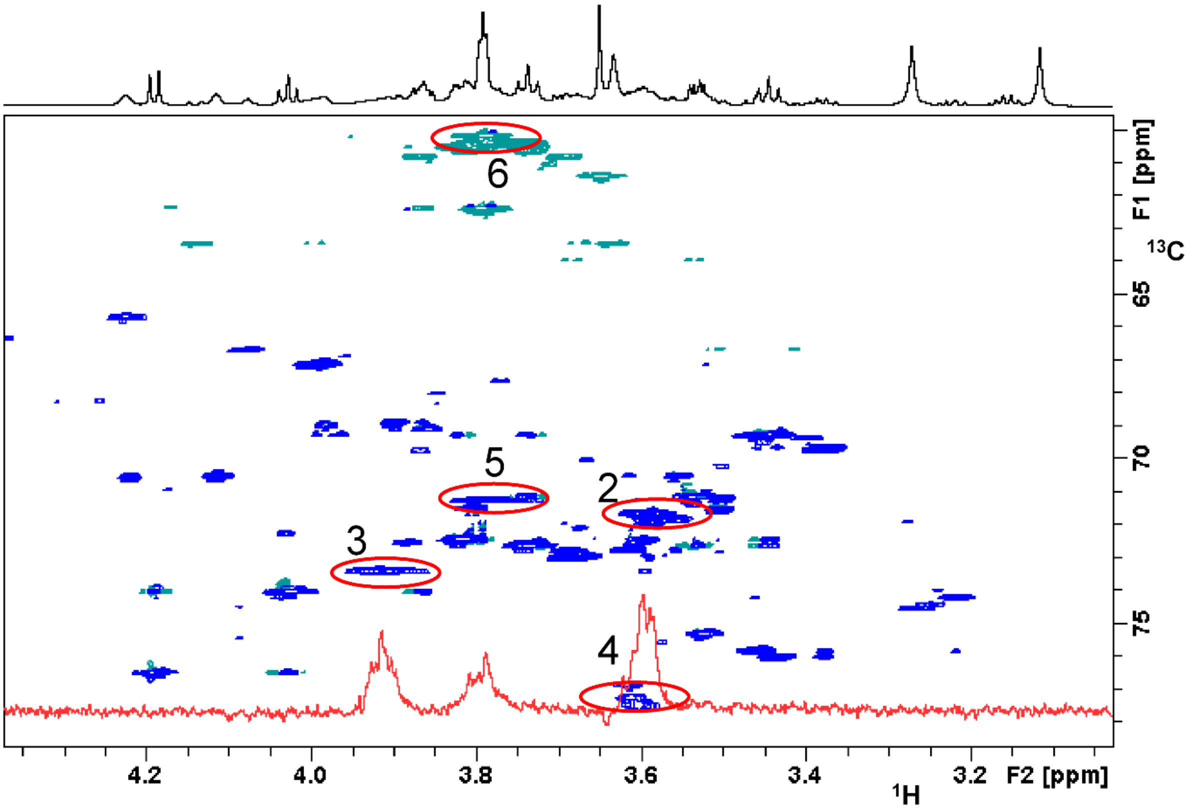

Fig. S3. Partial 800 MHz 2D ^{1}H, ^{13}C HSQC NMR spectra of HWE unburied tea highlighting the signals of amylopectin. The red trace represents a 1D CSSF-TOCSY[1] spectrum obtained by selective excitation of H-1 signal at 5.33 ppm and a mixing time of 120 ms. The ^{1}H/^{13}C chemical shifts of amylopectin are in excellent agreement with those of the middle unit of a Methyl α-D maltotrioside of an amylopectin.[2]

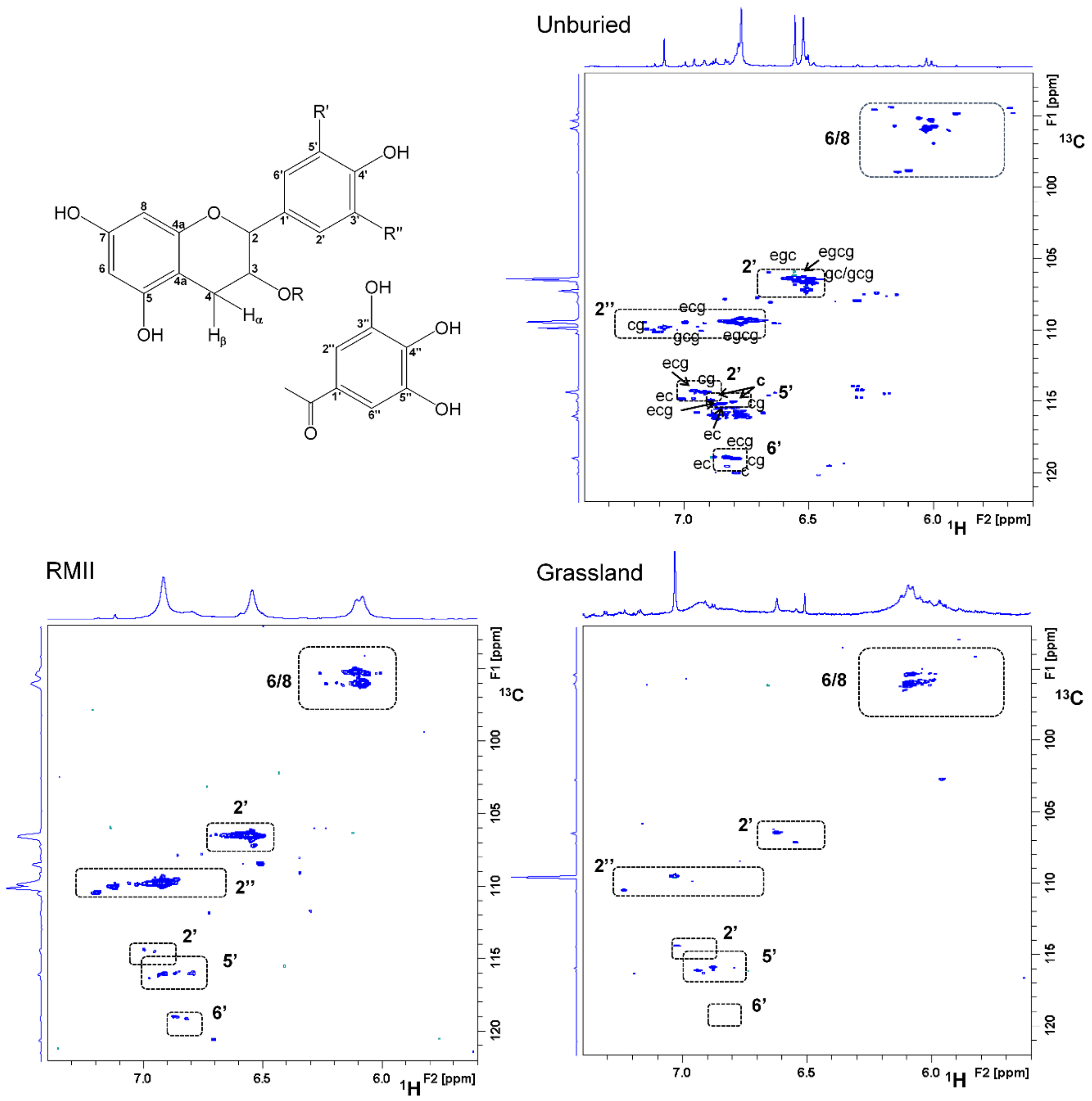

Fig. S4. Catechin region of the 800 MHz 2D ^{1}H, ^{13}C HSQC NMR spectra of HWE green tea from individual sites. The spectrum of the woodland buried sample is very similar to that of the grassland sample. The RMI sample yielded a spectrum similar to RMII, but with much reduced signal intensities.

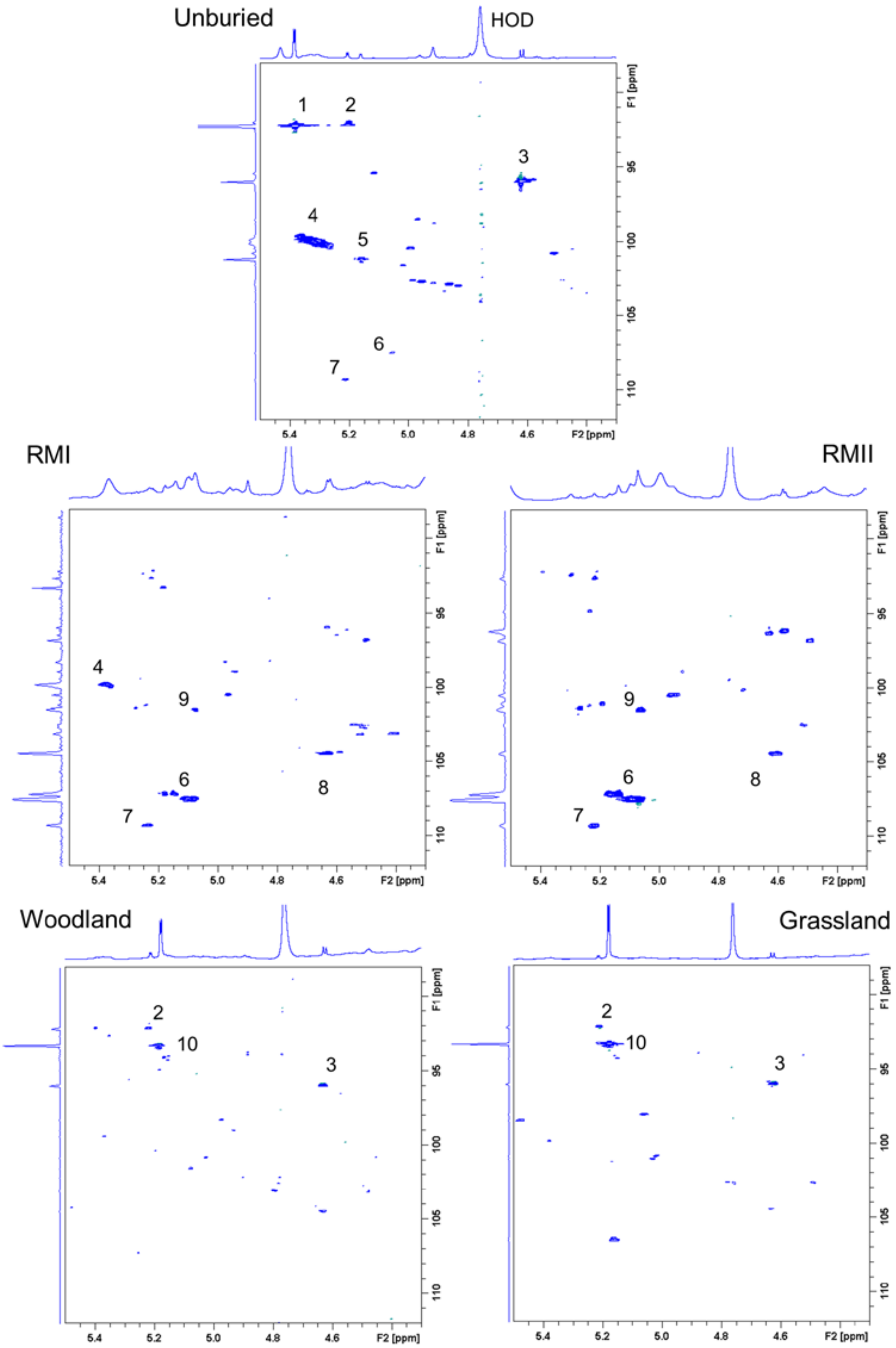

Fig. S5. Anomeric regions of the 800 MHz 2D ^{1}H, ^{13}C HSQC NMR spectra of HWE green tea from individual sites. Key to the assignments: **1**: sucrose (α-D-Glc), **2**: α-D-Glc, **3**: β-D-Glc, **4**: amylopectin, **5**: 2-O-(β-L-Arabinopyranosyl)-myo-inositol, **6**: Arabinogalactan (1,5-α-Ara*f*), **7**: Arabinogalactan (Terminal-α-Ara*f*), **8**: Arabinogalactan (β-Gal*p*), **9**: Arabinogalactan (α-Rha*f*), **10**: trehalose.

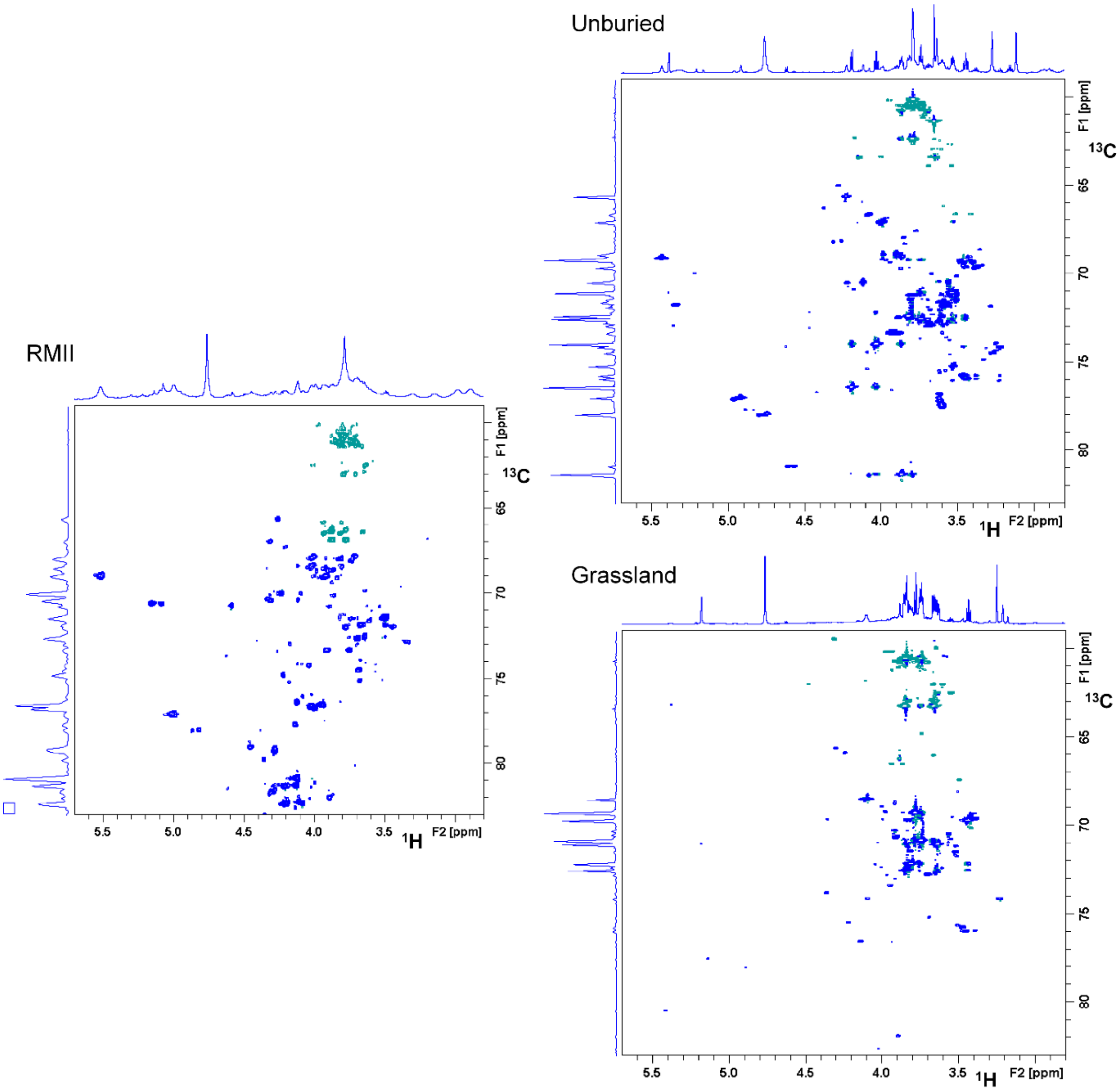

Fig. S6. Carbohydrate region of the 800 MHz 2D ^{1}H, ^{13}C HSQC NMR spectra of HWE green tea from individual sites. The unburied tea contains signals of sucrose, α/β-D-Glc, amylopectin, 2-O-(β-L-Arabinopyranosyl)-myo-inositol. The grassland spectrum is dominated by the signals mannitol and trehalose, while the RMII spectrum contains numerous signals of α-Ara*f*, β-Gal*p* and α-Rha*f* from arabinogalactan (CH_3 signals of α-Rha*f* appear at 1.25/16.5 and 1.29/16.8 ppm ($^{1}H/^{13}C$). The spectrum of the woodland buried sample is very similar to that to the grassland sample. The RMI sample yielded a spectrum similar to RMII, with less intense signals.

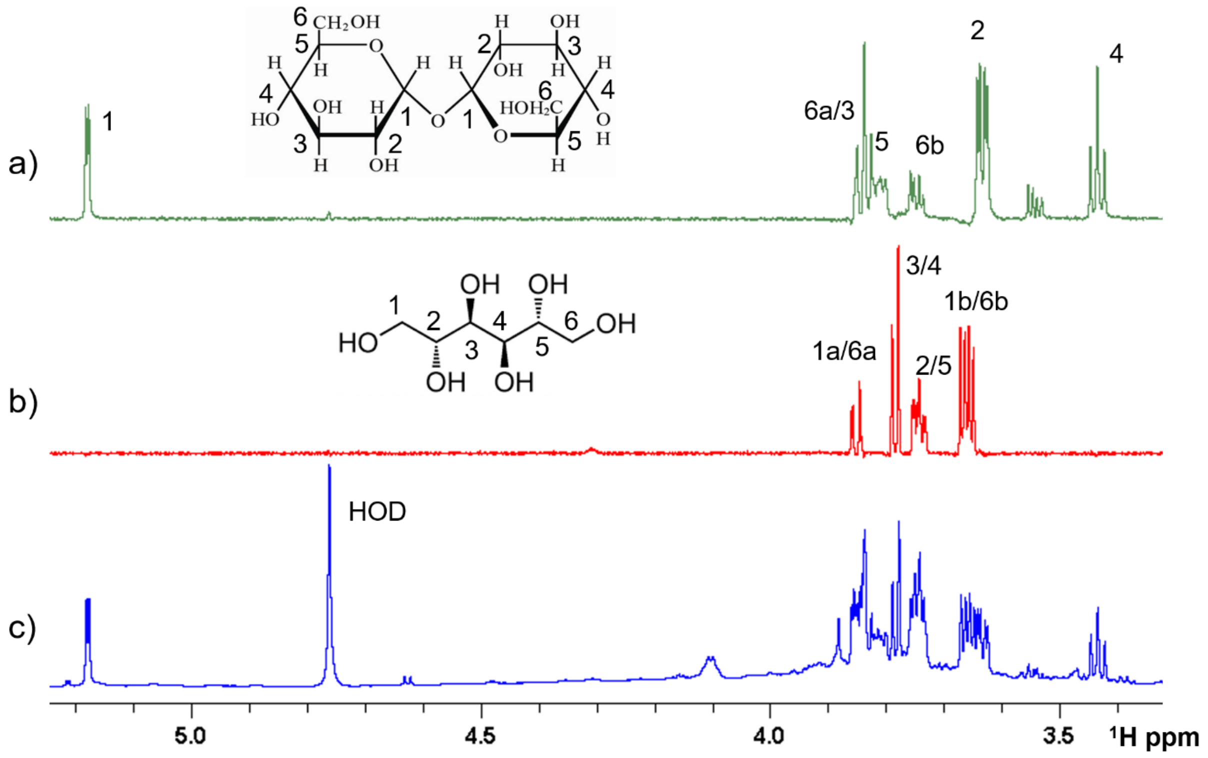

Fig. S7. 800 MHz 1D CSSF-TOCSY[1] spectra of (a) trehalose and (b) mannitol of the HWE tea buried in grassland; (c) shows the 1D ^{1}H spectrum. The carrier was positioned at the chemical shift of H-1b/6b (mannitol) or H-2 (trehalose).

Section S3: ^{1}H NMR spectra of buried and unburied green tea extracts used for PCA

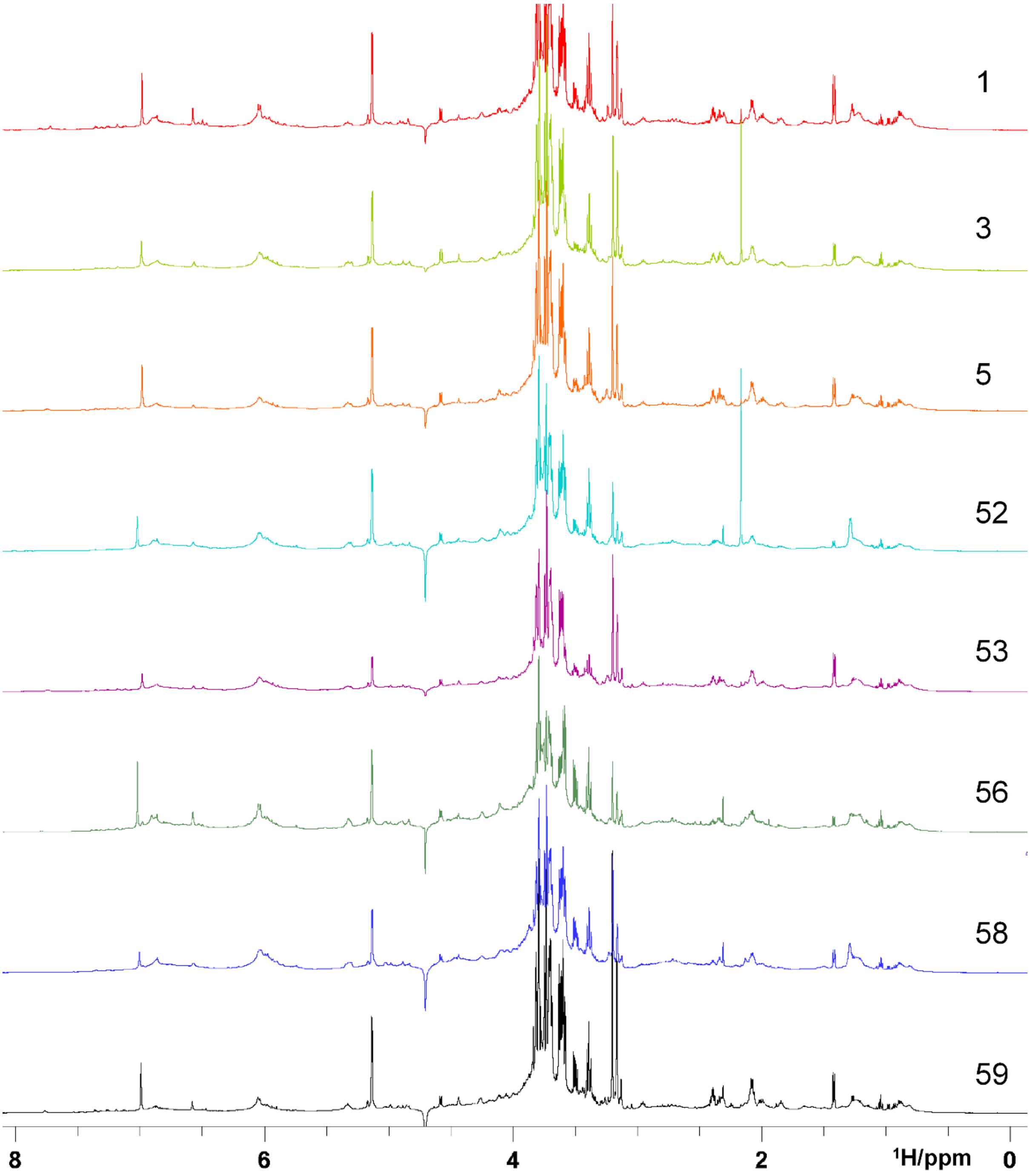

Fig. S8: 600 MHz ^{1}H NMR spectra of HWE green tea buried at the woodland site. Numbers at the right hand side represent the coding for the tea bags used.

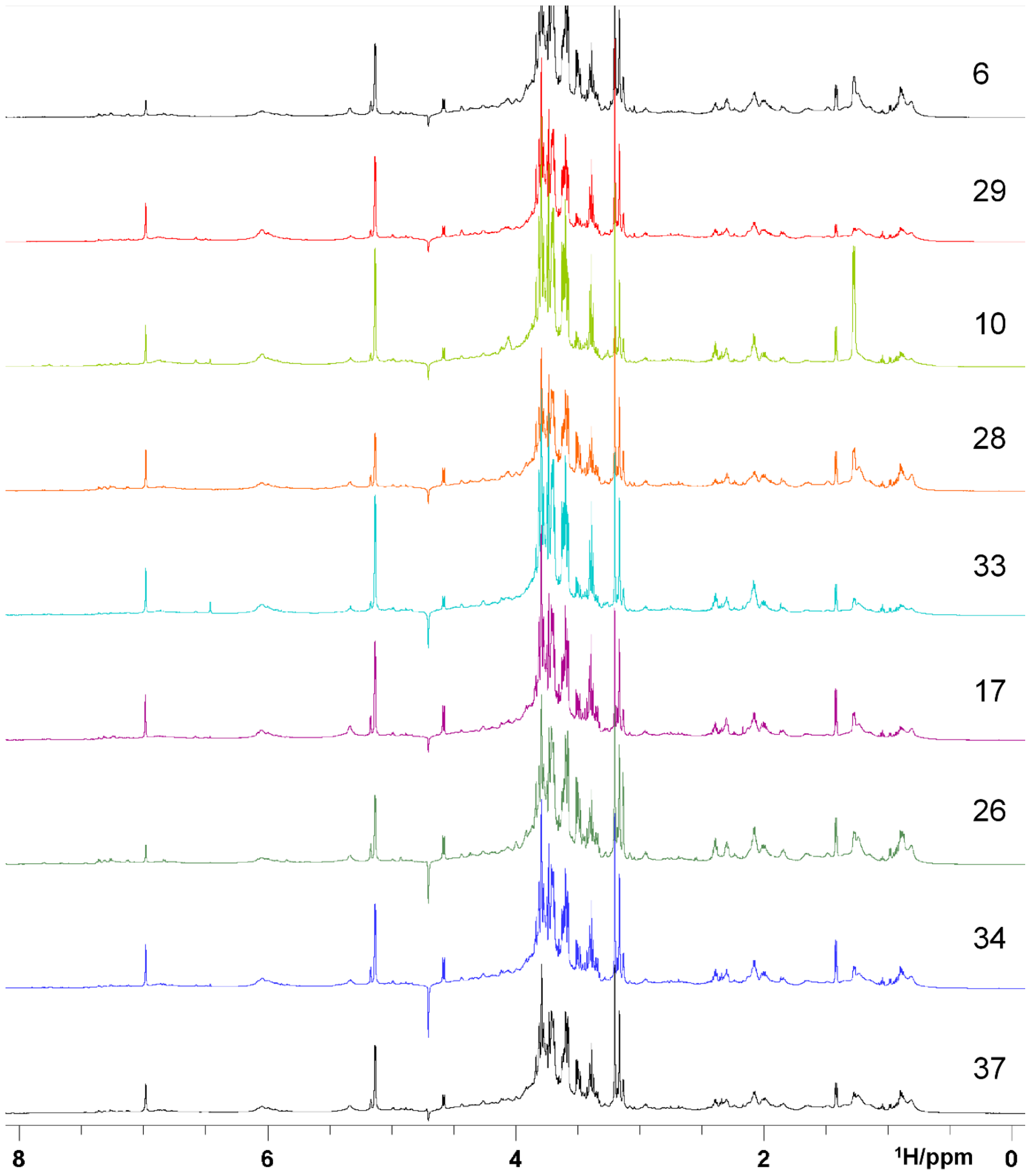

Fig. S9. 600 MHz ^{1}H NMR spectra of HWE green tea buried at the grassland site. Numbers at the right hand side represent the coding for the tea bags used.

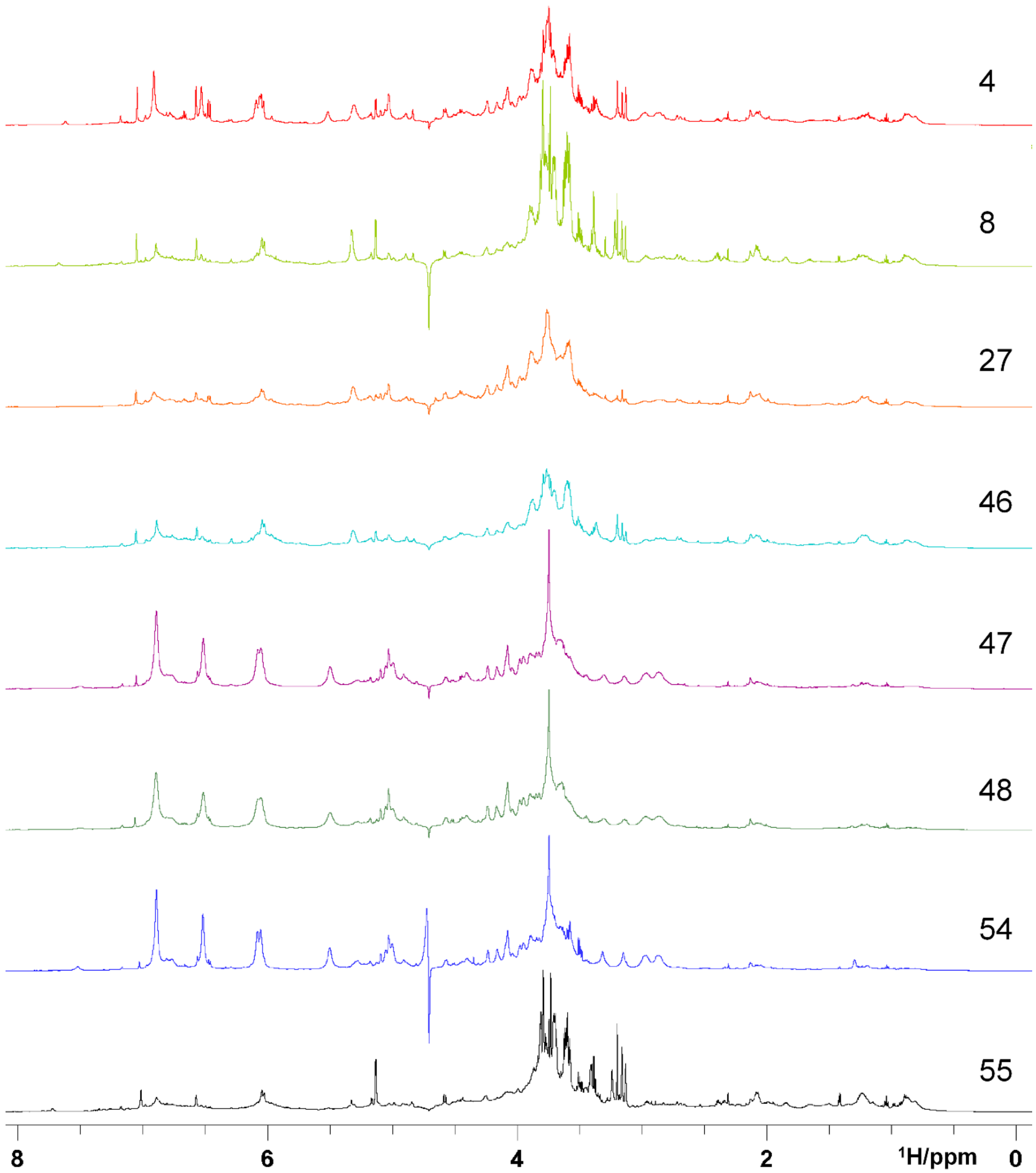

Fig. S10. 600 MHz ^{1}H NMR spectra of HWE green tea buried at the damaged peat bog site (RMI). Numbers at the right hand side represent the coding for the tea bags used.

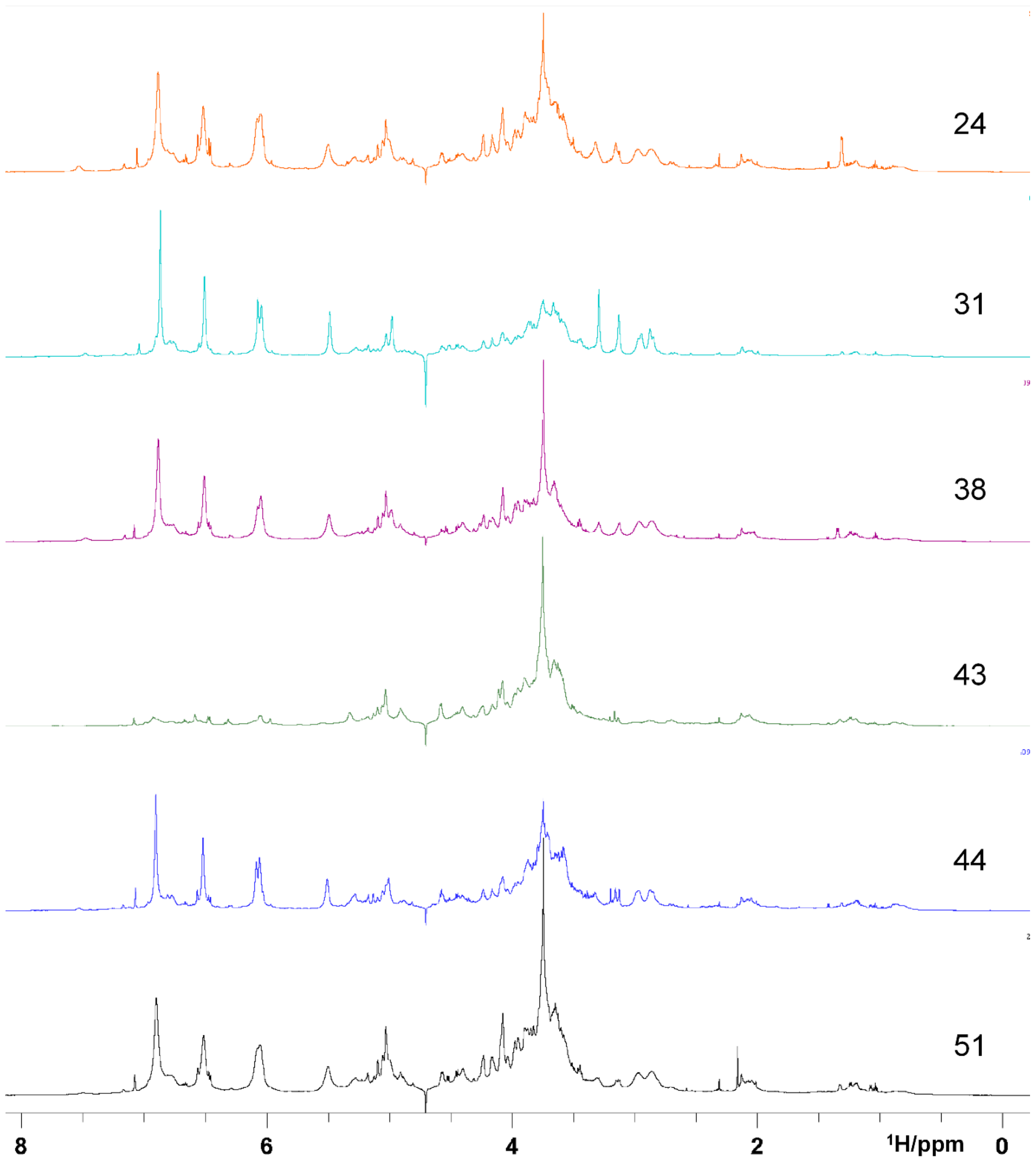

Fig. S11. 600 MHz ^{1}H NMR spectra of HWE green tea buried at the peat bog site under restoration (RMII). Numbers at the right hand side represent the coding for the tea bags used.

Section S4. Analysis of ^{1}H relaxation times of the HWE green tea samples

Table SIII. T_1 relaxation times [s] of protons resonating in the three spectral regions.[a]

Sample/region	8.0-4.9 ppm [%][b]	4.6-3.2 ppm [%][b]	3.2-0.8 ppm [%][b]	All regions [%][b]
Unburied tea, G11	0.88± 0.13 [-]	0.78 ± 0.14 [-]	0.47 ± 0.19 [-]	0.72 ± 0.23 [-]
RMII, G24	0.51± 0.05 [**-42**]	0.54 ± 0.08 [**-31**]	0.39 ± 0.04 [**-17**]	0.47 ± 0.08 [**-35**]
RMI, G27	0.39± 0.07 [**-56**]	0.44 ± 0.12 [**-44**]	0.33 ± 0.03 [**-30**]	0.39 ± 0.09 [**-46**]
Woodland, G1	0.62± 0.19 [**-30**]	0.74 ± 0.13 [**-5**]	0.54 ± 0.07 [**+15**]	0.64 ± 0.15 [**-11**]
Grassland, G33	0.44± 0.16 [**-50**]	0.72 ± 0.17 [**-8**]	0.47 ± 0.08 [**0**]	0.53 ± 0.18 [**-26**]

[a] Standard deviation across signals within the spectral range stated.

[b] The numbers in brackets represent a change in % relative to the unburied sample.

Table SIV. Fast T_2 component of the ^{1}H relaxation times /ms.[a]

Sample/region	8.0-4.9 ppm [%][b]	4.6-3.2 ppm [%][b]	3.2-0.8 ppm [%][b]	All regions [%][b]
Unburied tea, G11	77 ± 30 [-]	61 ± 35 [-]	24 ± 7 [-]	55 ± 35 [-]
RMII, G24	32 ± 14 [**-58**]	30 ± 10 [**-51**]	22 ± 10 [**-8**]	28 ± 12 [**-49**]
RMI, G27	19 ± 4 [**-75**]	24 ± 7 [**-61**]	20 ± 4 [**-17**]	21 ± 5 [**-62**]
Woodland, G1	62 ± 14 [**-19**]	43 ± 17 [**-30**]	26 ± 8 [**+8**]	44 ± 20 [**-20**]
Grassland, G33	45 ± 10 [**-42**]	44 ± 31 [**-28**]	24 ± 7 [**0**]	39 ± 20 [**-29**]

[a] Standard deviation across signals within the spectral range stated.

[b] The numbers in brackets represent a change in % relative to the unburied sample.

Table SV. Slow T_2 component of the ^{1}H relaxation times /ms.[a]

Sample/region	8.0-4.9 ppm [%][b]	4.6-3.2 ppm [%][b]	3.2-0.8 ppm [%][b]	All regions [%][b]
Unburied tea, G11	353 ± 143 [-]	397 ± 93 [-]	283 ± 170 [-]	345 ± 144 [-]
RMII, G24	211 ± 114 [**-40**]	261 ± 44 [**-34**]	231 ± 70 [**-18**]	233 ± 82 [**-32**]
RMI, G27	97 ± 26 [**-73**]	217 ± 134 [**-45**]	154 ± 32 [**-46**]	156 ± 118 [**-55**]
Woodland, G1	357 ± 196 [**+1**]	467 ± 116 [**+18**]	306 ± 101 [**+8**]	369 ± 163 [**+7**]
Grassland, G33	221 ± 141 [**-37**]	358 ± 101 [**-10**]	242 ± 59 [**-14**]	279 ± 105 [**-19**]

[a] Standard deviation across signals within the spectral range stated.

[b] The numbers in brackets represent a change in % relative to the unburied sample.

The results of the relaxation analysis are summarised in the Tables SIII-SV, where the average ^{1}H relaxation times corresponding to three spectral regions and the whole spectrum are given.

The stated standard deviations indicate the spread of the relaxation times across resonances within the stated ppm range, rather than the accuracy of the measurements. It should be emphasized that the ^{1}H relaxation times obtained from spectra of mixtures with abundant signal overlap can at best be interpreted in a qualitative manner. When a mixture consists of large and small molecules of different concentration, as is the case in this sample set, such analysis is further complicated. This became particularly apparent for the T_2 relaxation data, where fitting with a mono exponential function yielded poor results. A bi-exponential analysis, making an allowance for fast and slow components to the T_2 relaxation, improved the quality of the fit considerably and allowed trends to be inferred.

Generally, the T_1 and T_2 relaxation times of buried samples shortened significantly relative to those of the unburied tea sample. The largest drop in T_1 relaxation times (obtained in a mono-exponential fit) were seen for the RMI sample, while least affected was the woodland sample, where the change varied for individual spectral regions (see Table SIII). Here small changes were obtained for the carbohydrate and aliphatic regions (-5 and +15% respectively) and the aromatic region showed the smallest decrease in T_1 values (-30%) amongst the buried samples. Taken together, as seen by the T_1 ^{1}H NMR data, the woodland and RMI sample contain the largest and the smallest proportion of small molecules, respectively.

The bi-exponential fit of the T_2 relaxation data across the whole sample set and the 8.0 – 0.8 ppm region yielded the fast and slow relaxation times of 37 ± 13 ms and 276 ± 86 ms, respectively. For both of these timescales the trends followed those seen for the T_1 data, with the woodland and RMI samples being the two extremities. Similar trends were seen for the three discussed spectral regions. For the woodland sample, the results were very close to those obtained for the unburied sample, despite large visual differences in the appearances of the two spectra. This indicates that, within the NMR detection limit, the woodland sample is a mixture, containing mostly small molecules, while the RMI sample contains larger molecules.

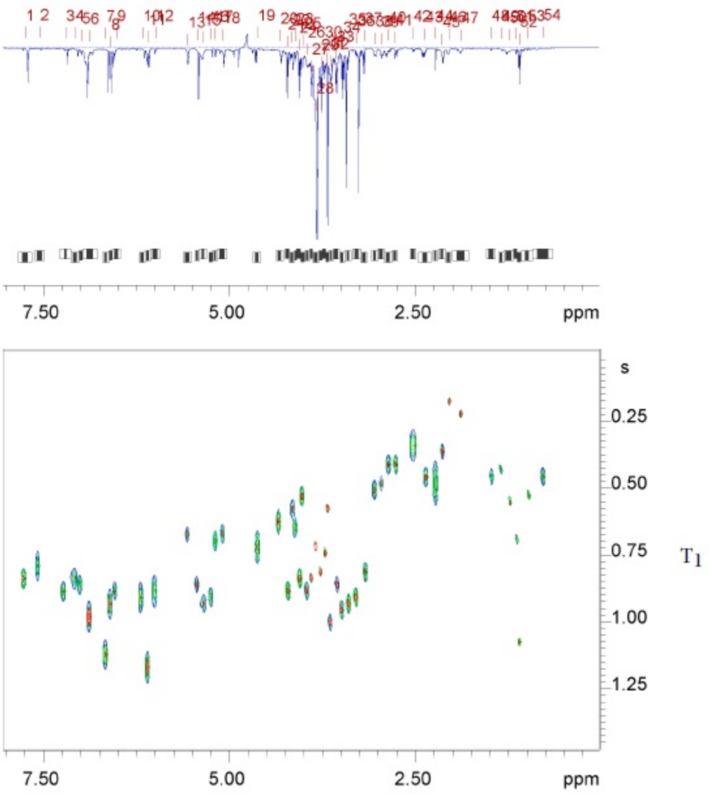

Fig. S12. 2D T_1 relaxation map of the HWE unburied green tea sample.

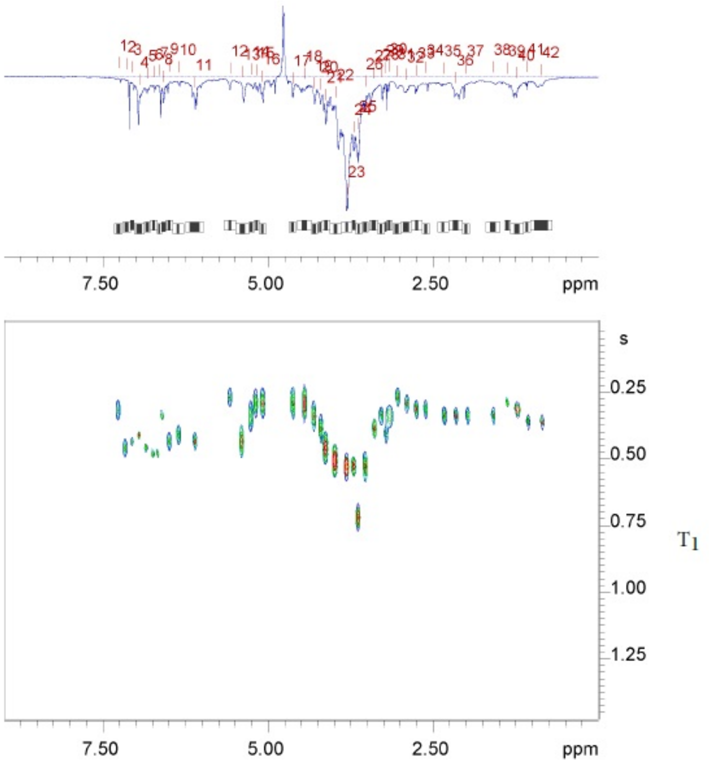

Fig. S13. 800 MHz 2D T_1 relaxation map of the HWE green tea sample buried in RMI.

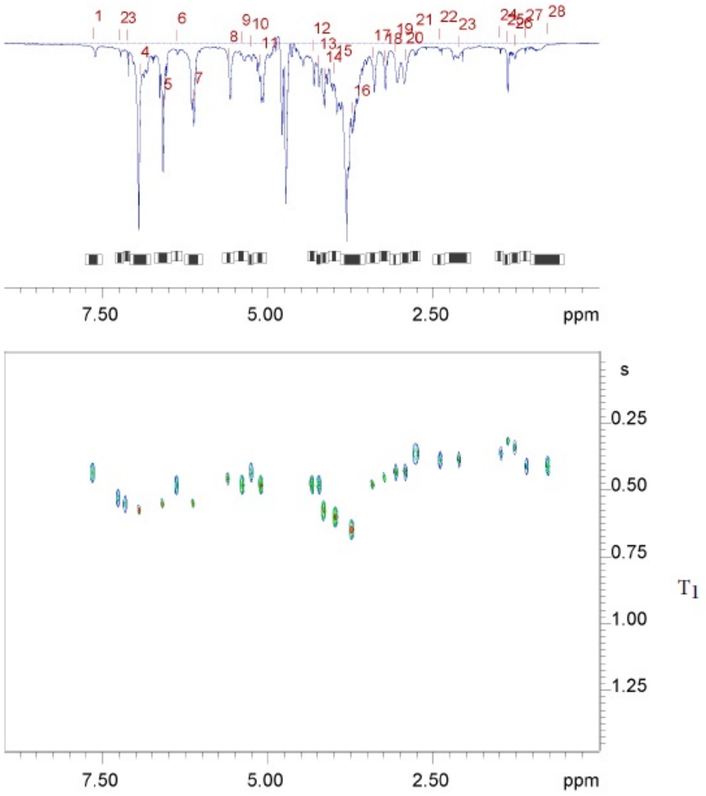

Fig. S14. 800 MHz 2D T_1 relaxation map of the HWE green tea sample buried in RMII.

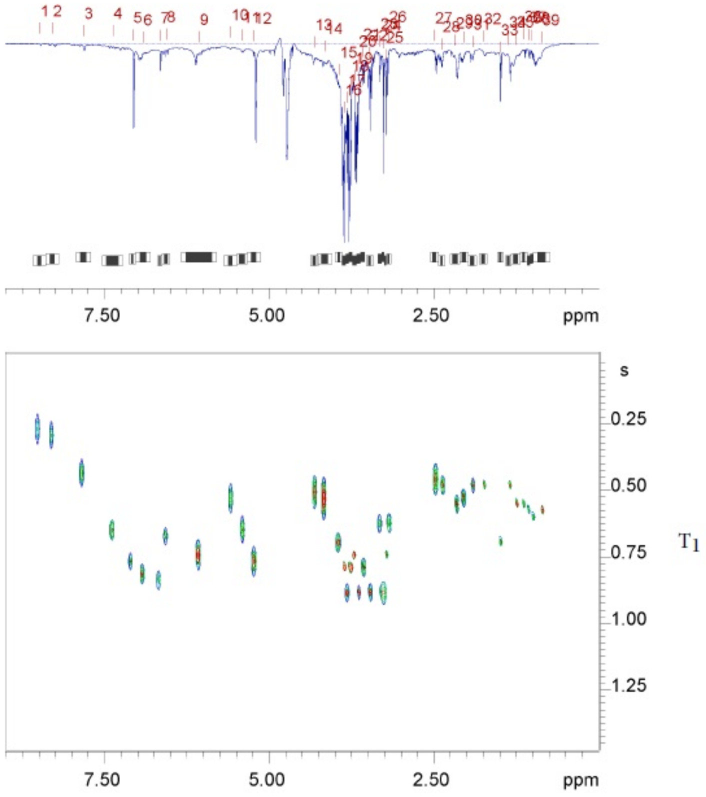

Fig. S15. 800 MHz 2D T_1 relaxation map of the HWE green tea buried in woodland.

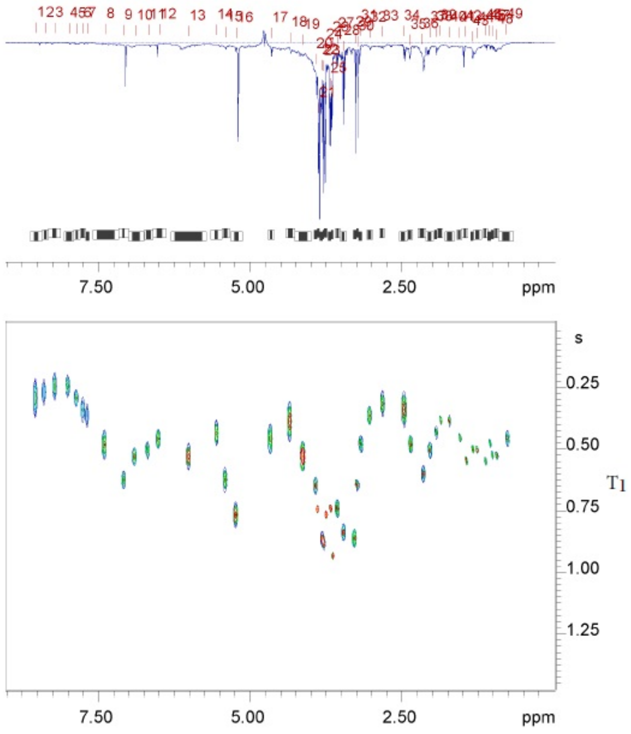

Fig. S16. 800 MHz 2D T_1 relaxation map of the HWE green tea buried in grassland.

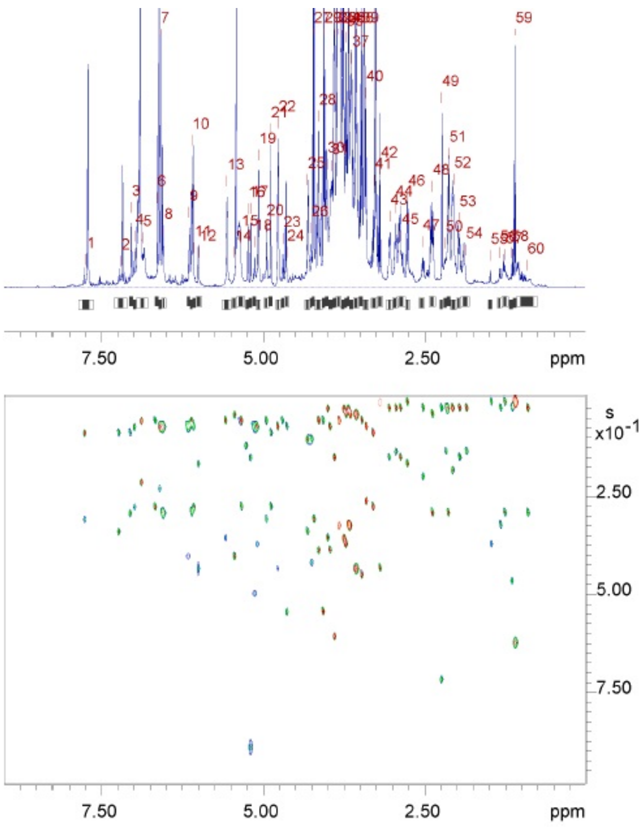

Fig. S17. 800 MHz 2D T_2 relaxation map of the HWE unburied green tea sample.

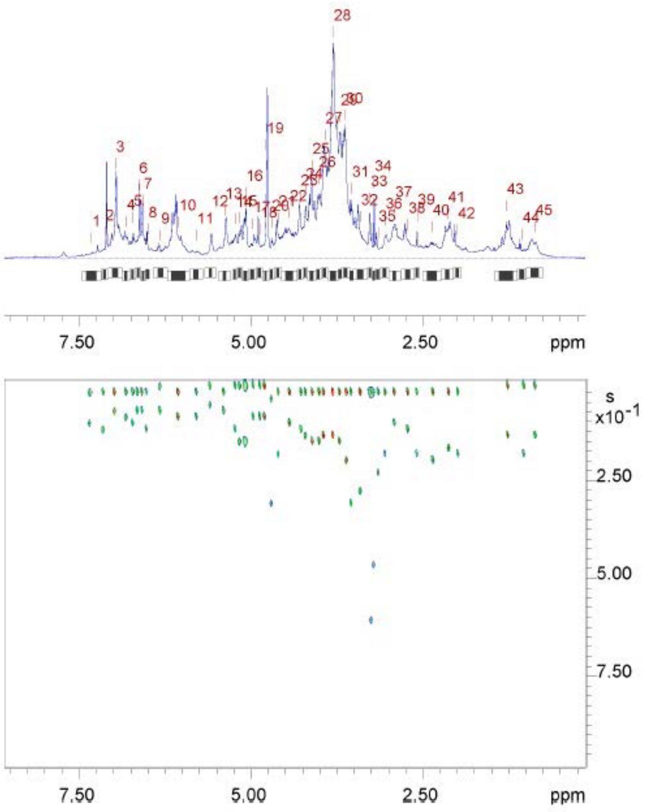

Fig. S18. 800 MHz 2D T_2 relaxation map of the HWE green tea buried in RMI.

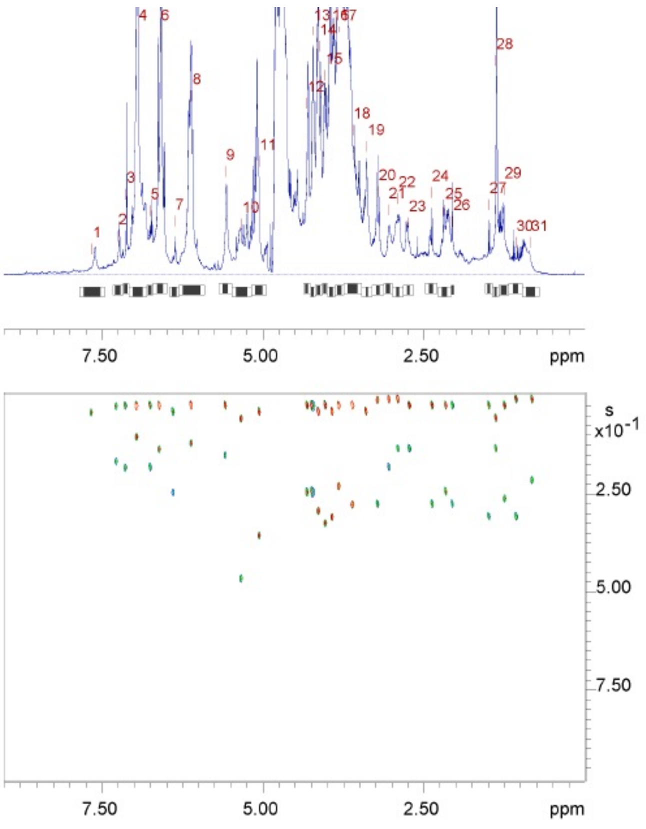

Fig. S19. 800 MHz 2D T_2 relaxation map of the HWE green tea buried in RMII.

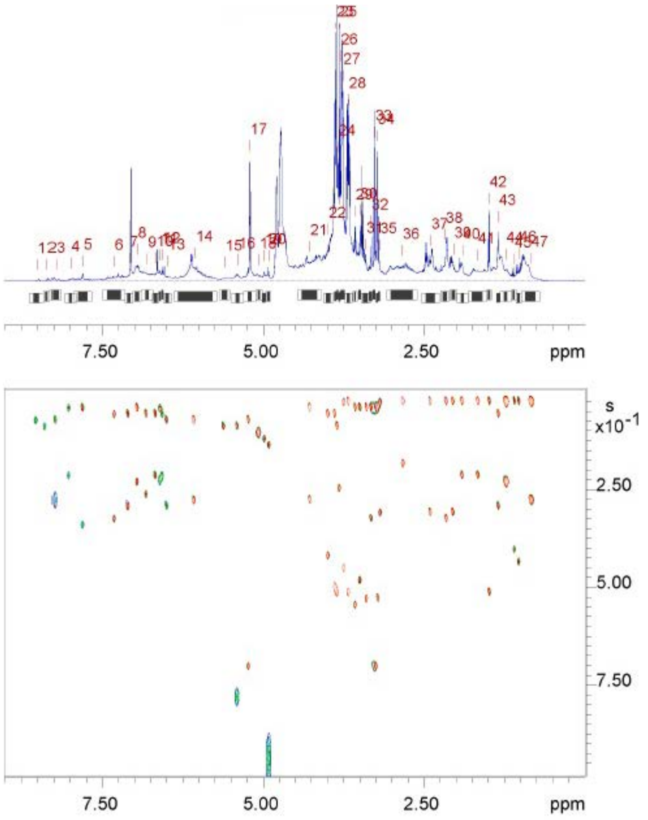

Fig. S20. 800 MHz 2D T_2 relaxation map of the HWE green tea buried in woodland.

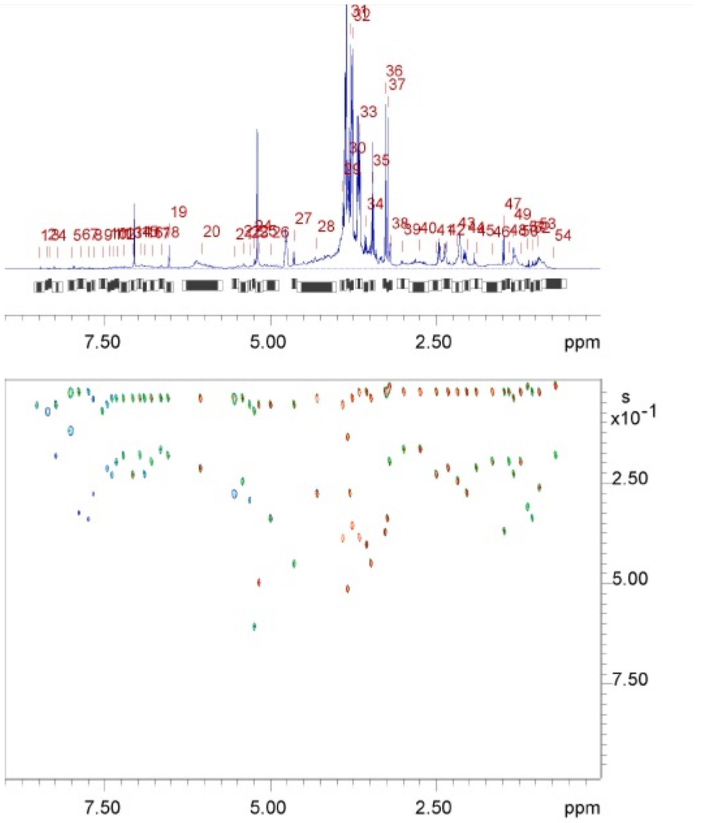

Fig. S21. 800 MHz 2D T_2 relaxation map of the HWE green tea buried in grassland.

Section S5. Analysis of DOSY spectra of the HWE green tea samples

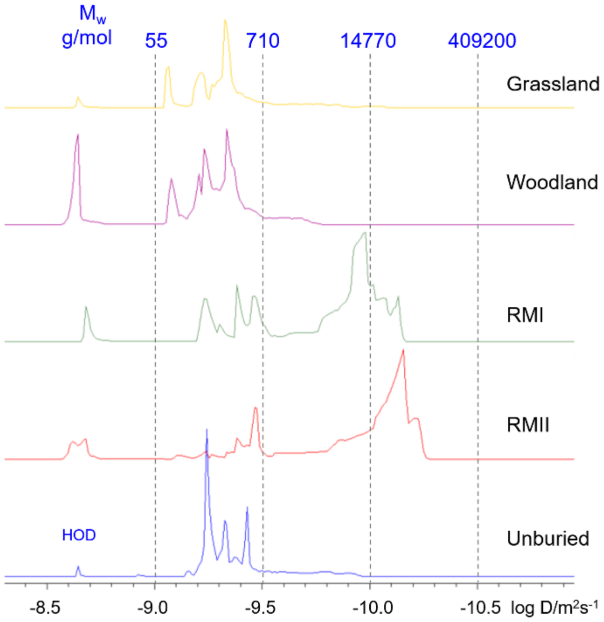

Fig. S22. The F_1 projection of 800 MHz 2D DOSY spectra of the HWE green tea samples. The diffusion coefficients presented on the logarithmic scale were converted into molecular weights.[3] Note: water presaturation caused some distortions of the HOD signal in the RMII spectrum but the signal still appeared at the expected position in the spectra.

Similarly to the relaxation data, the DOSY spectra of complex mixtures containing overlapping signals must be interpreted with caution as only averaged values are returned from the fitted data. In addition, the conversion of diffusion coefficients into molecular weights is an approximate process as differing molecular shapes affect the obtained values. With these caveats in mind, the DOSY spectra provided useful insights into the decomposition processes taking place in the buried samples.

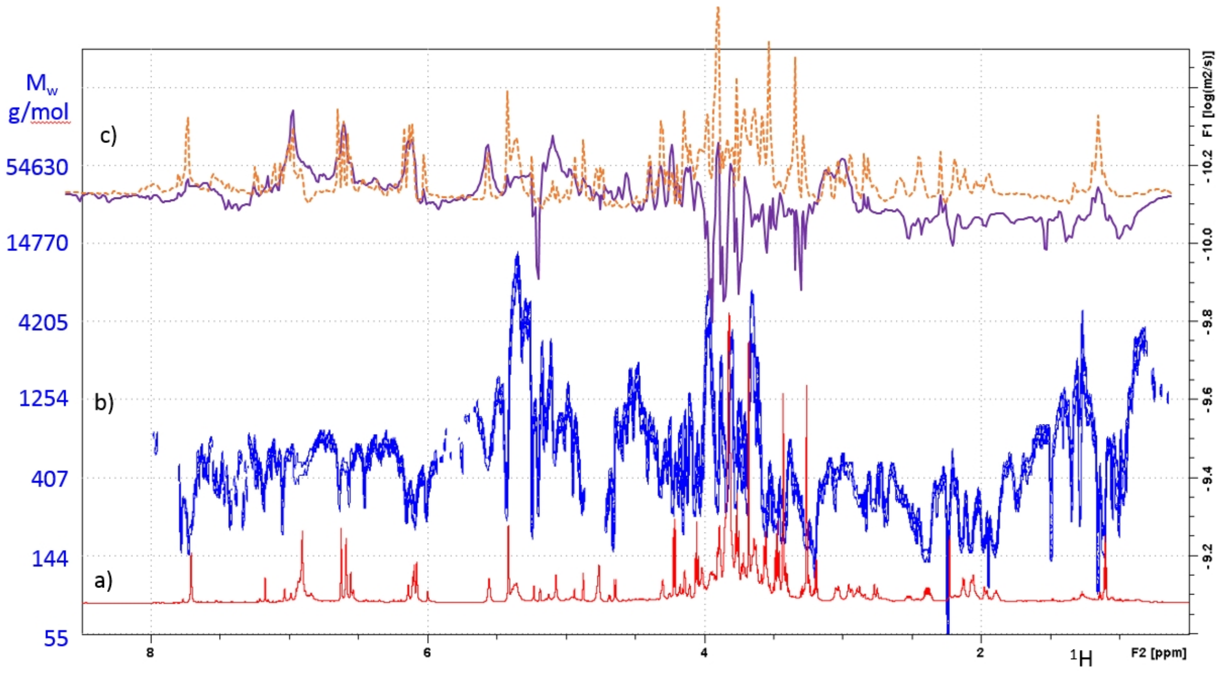

Fig. S23. DOSY analysis of the HWE unburied green tea. a) 800 MHz ^{1}H NMR spectrum b) 800 MHz DOSY spectrum c) PC1 (full line) and PC2 (dashed line) loadings plot from the PCA analysis of all samples including the unburied green tea samples. Note that the PC1 loadings are (except for the opposite sign) very similar to those obtained for the analysis of only the buried green tea samples (Fig. S27). This reflects the fact that the group separation along PC1 is essentially identical in both analyses.

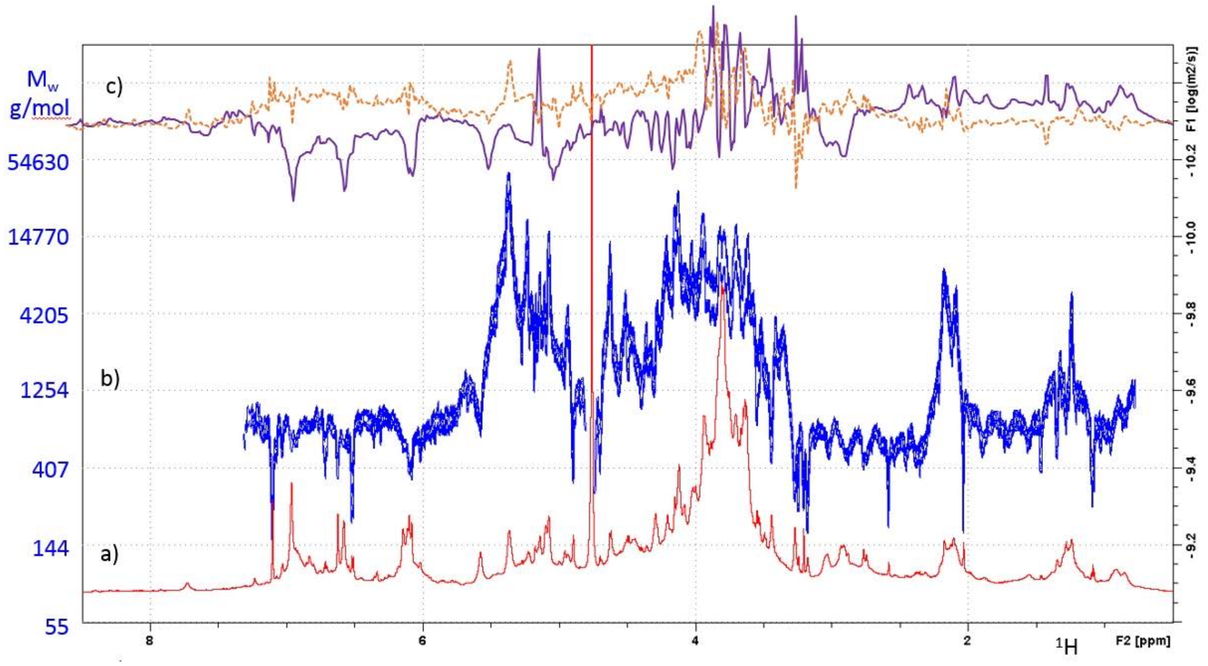

Fig. S24. DOSY analysis of the HWE green tea buried in the RMI site. a) 800 MHz ^{1}H NMR spectrum b) 800 MHz DOSY spectrum c) PC1 (full line) and PC2 (dashed line) loadings plot from the PCA analysis of all buried green tea samples only.

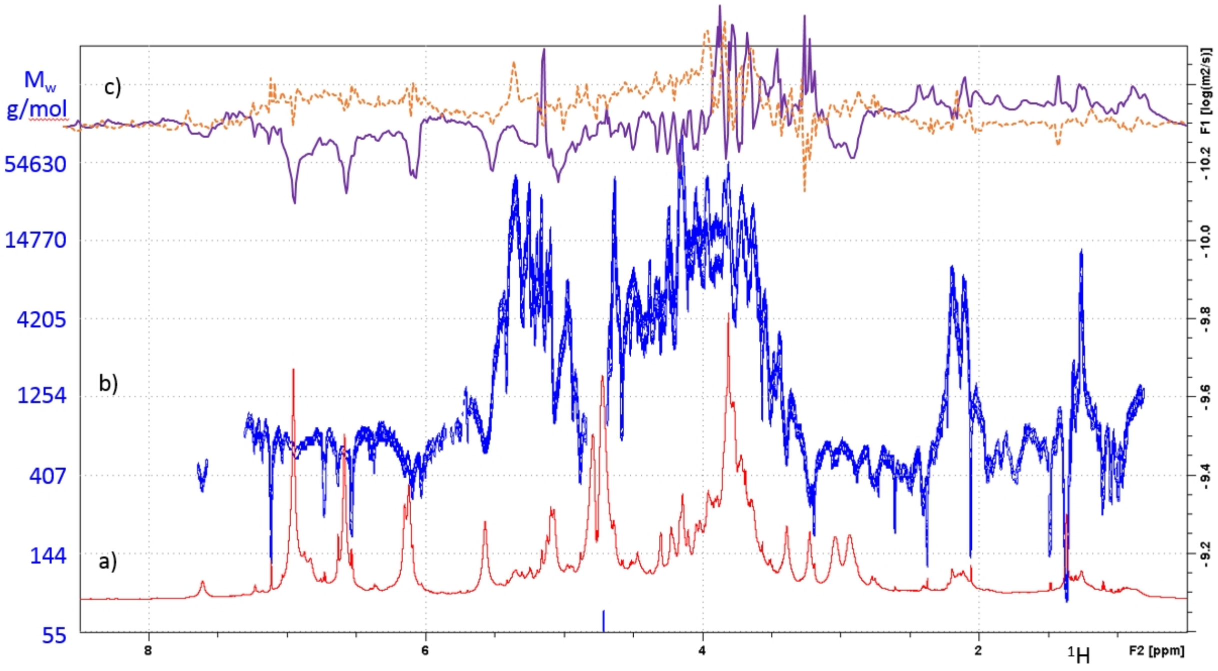

Fig. S25. DOSY analysis of the HWE green tea buried in the RMII site. a) 800 MHz ^{1}H NMR spectrum b) 800 MHz DOSY spectrum c) PC1 (full line) and PC2 (dashed line) loadings plot from the PCA analysis of all buried green tea samples only.

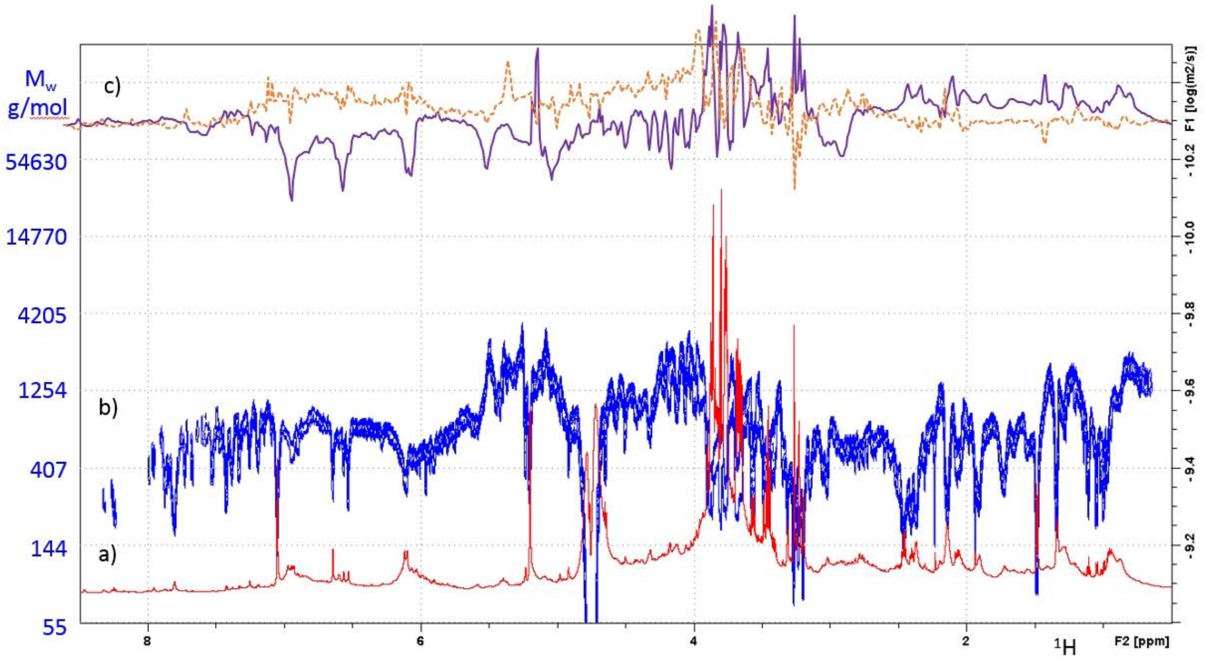

Fig. S26. Analysis of the HWE green tea buried in the woodland site. a) 800 MHz ^{1}H NMR spectrum b) 800 MHz DOSY spectrum c) PC1 (full line) and PC2 (dashed line) loadings plot from the PCA analysis of all buried green tea samples only.

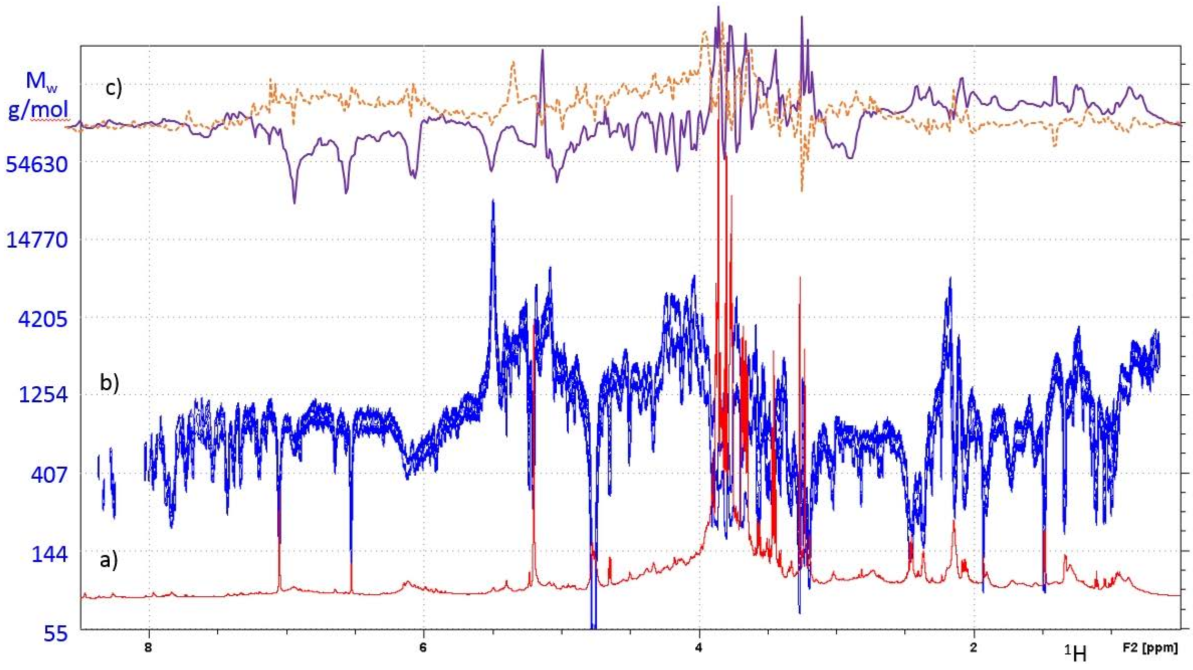

Fig. S27. Analysis of the HWE green tea buried in the grassland site. a) 800 MHz ^{1}H NMR spectrum b) 800 MHz DOSY spectrum c) PC1 (full line) and PC2 (dashed line) loadings plot from the PCA analysis of all buried green tea samples only.

Section S6. PCA of ^{1}H NMR spectra of buried and unburied HWE green tea.

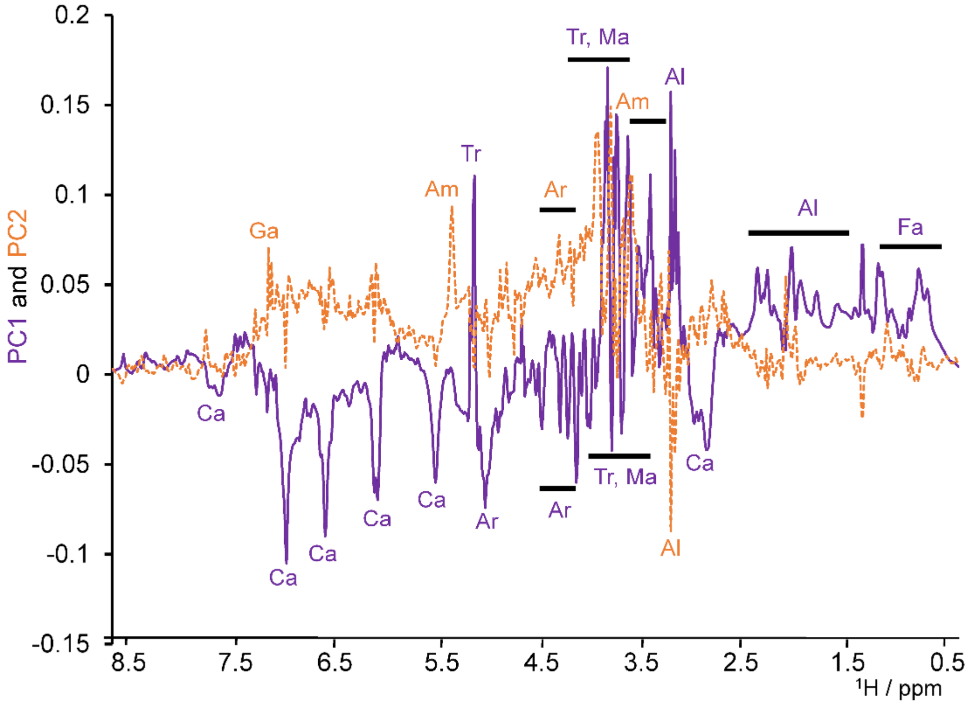

Fig. S28. PCA loadings plots associated with the score plot shown in Fig. 3: PC1 (purple) and, PC2 (brown). The compounds contributing most to the definition of PC1 are indicated as Ca - catechins, Tr - trehalose, Ma - mannitol, Al - aliphatic molecules, Am - amylopectin, Ar - arabinogalactan, Ga - gallic acid, Fa - fatty acids.

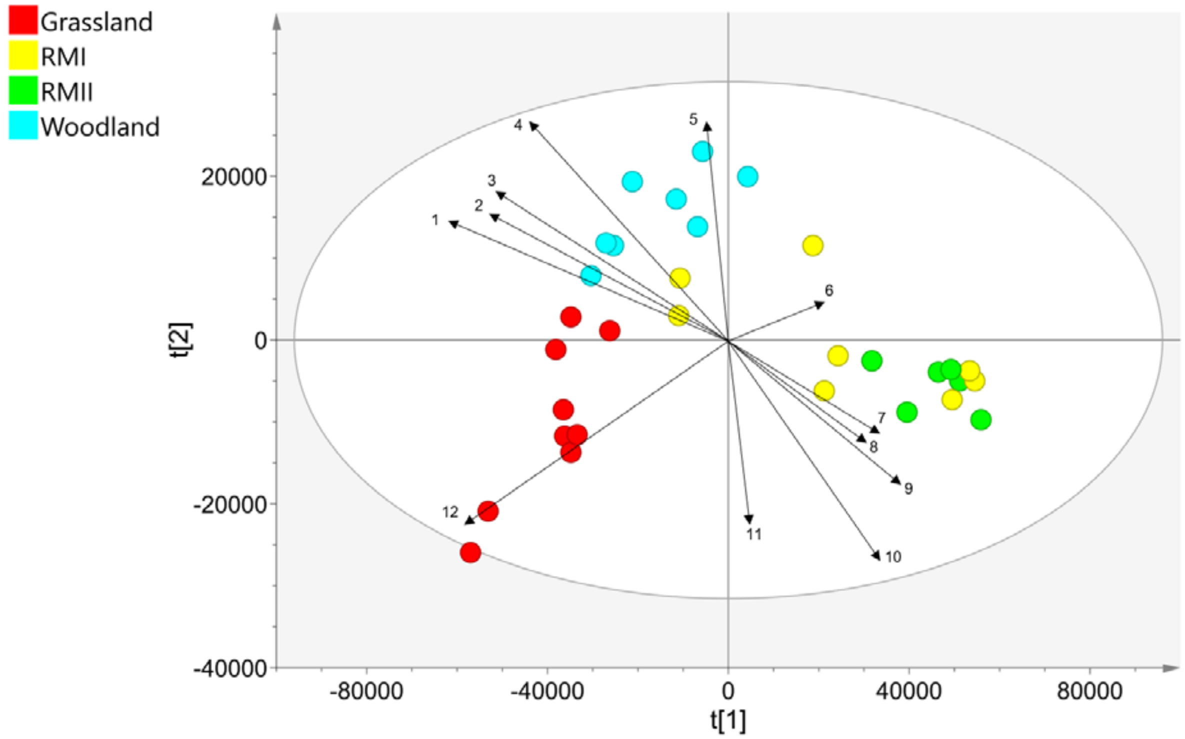

Number	Bin centered at δ/ppm*	Assignment
1	3.787	Trehalose/mannitol
2	3.697	Trehalose/mannitol
3	3.807	Trehalose/mannitol
4	3.727	Trehalose/mannitol
5	2.157	Aliphatic
6	6.047	Catechin
7	6.517	Catechin
8	6.507	Catechin
9	6.877	Catechin
10	6.887	Catechin
11	3.667	Trehalose/mannitol
12	3.197	Aliphatic

*the width of bins is 0.01 ppm

Fig. S29. PLS-DA score plot based on the 1D ^{1}H 600 MHz NMR spectra of the HWE buried green tea samples without unburied green tea (R^2X = 0.659, R^2Y = 0.507 and Q^2 = 0.263). The Hotelling's T^2 ellipse represents 95% confidence interval. The key indicates the colour coding for each sample site. The PC1/PC2 loadings are overlaid in the form of biplots, indicating the spectral regions that contributed most to sample separation. The assignment is provided in the Table above.

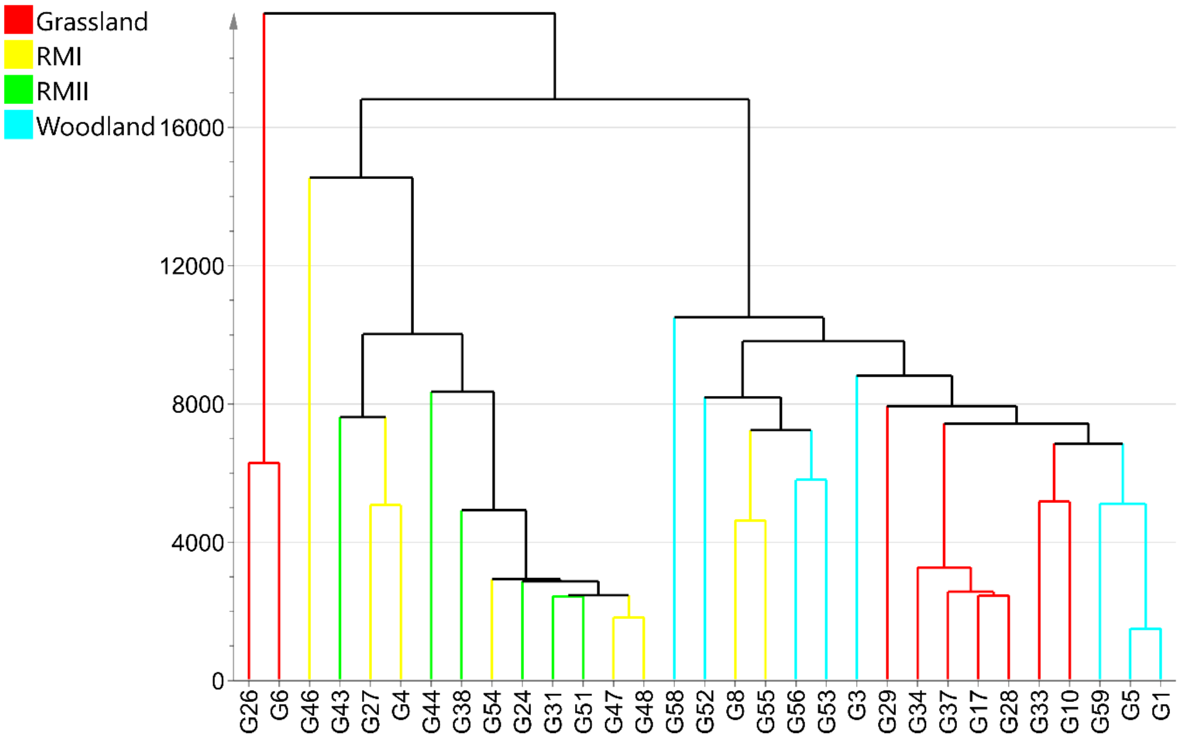

Fig. S30. HCA dendrogram based on the PLS-DA of 1D ^{1}H 600 MHz NMR spectra of the HWE buried green tea samples without unburied green tea. The key indicates the colour coding for each sample site.

Section 7. FT-ICR-MS analysis of buried and unburied HWE green tea.

Table SVIII. Statistics for the FT-ICR-MS spectra of green tea samples.

**Site (number of samples)**	**Average number of picked peaks (S/N > 5) [%][a]**	**% of peaks assigned[b] [relative std / %][a]**
Unburied (6)	1111 [± 17]	83 [± 0.4]
Red Moss II (6)	611 [± 14]	81 [± 1]
Red Moss I (6)	788 [± 39]	82 [± 1]
Red Moss I (3)[c]	1033 [± 23]	82 [± 2]
Red Moss I (3)[d]	543 [± 9]	82 [± 1]
Grassland (12)	1538 [± 17]	86 [± 2]
Woodland (8)	1543 [± 12]	83 [± 2]

[a] relative standard deviation for the samples in each site; [b] error threshold ± 0.5 ppm; ; [c] RMI samples with a large number of peaks; [d] RMI samples with a low number of peaks.

Table SIX. Average assignment statistics for the FT-ICR-MS spectra of green tea samples.

**Site (number of samples)**	**Total number of unique formulae assigned**	**Average number of formulae assigned [relative std /%][a]**
Unburied (6[b])	834	595 [± 12]
Red Moss I (6)	1079	524 [± 46]
Red Moss I (3)[c]	1010	721 [± 22]
Red Moss I (3)[d]	437	327 [± 9]
Red Moss II (6)	620	372 [± 18]
Grassland I (8)	1667	1040 [± 18]
Woodland (8)	1675	1052 [± 11]

[a] relative standard deviation for the samples in each site; [b]three measurements were duplicates of one sample; [c] RMI samples with a large number of peaks; [d] RMI samples with a low number of peaks.

Table SX. Summary of the number of compounds and % relative to unburied tea for each intercept from the UpSet plot given in Fig. 4.

**Sample type (number of)**	**Intercept(s)**	**Number of formulas**	**% relative to the unburied tea**
Produced in grassland and woodland only	I_1	402	49.8
**Preserved in every environment[a]**	$\mathbf{I_2}$	351	43.5
Produced in grassland, woodland and RMI	I_3	329	40.8
**Produced exclusively in grassland[a]**	$\mathbf{I_4}$	294	36.4
**Removed in every environment[a]**	$\mathbf{I_5}$	222	27.5
**Produced exclusively in woodland[a]**	$\mathbf{I_6}$	220	27.3
**Produced in every environment[a]**	$\mathbf{I_7}$	103	12.8
Removed in RMI and RMII	I_8	78	9.7
Removed exclusively in RMII	I_9	66	8.2
Produced exclusively in RMI	I_{10}	57	7.1
Produced exclusively in RMII	I_{15}	23	2.9
Removed exclusively in RMI	I_{24}	3	0.4
Not produced in RMII	$I_1 + I_3 + I_4 + I_6$	1247	154.3
Produced in grassland or woodland only	$I_1 + I_4 + I_6$	918	113.8
**Produced in RMI or RMII only[a]**	$\mathbf{I_{10} + I_{12} + I_{15}}$	120	14.9

[a] Highlighted intersections are discussed in the main paper.

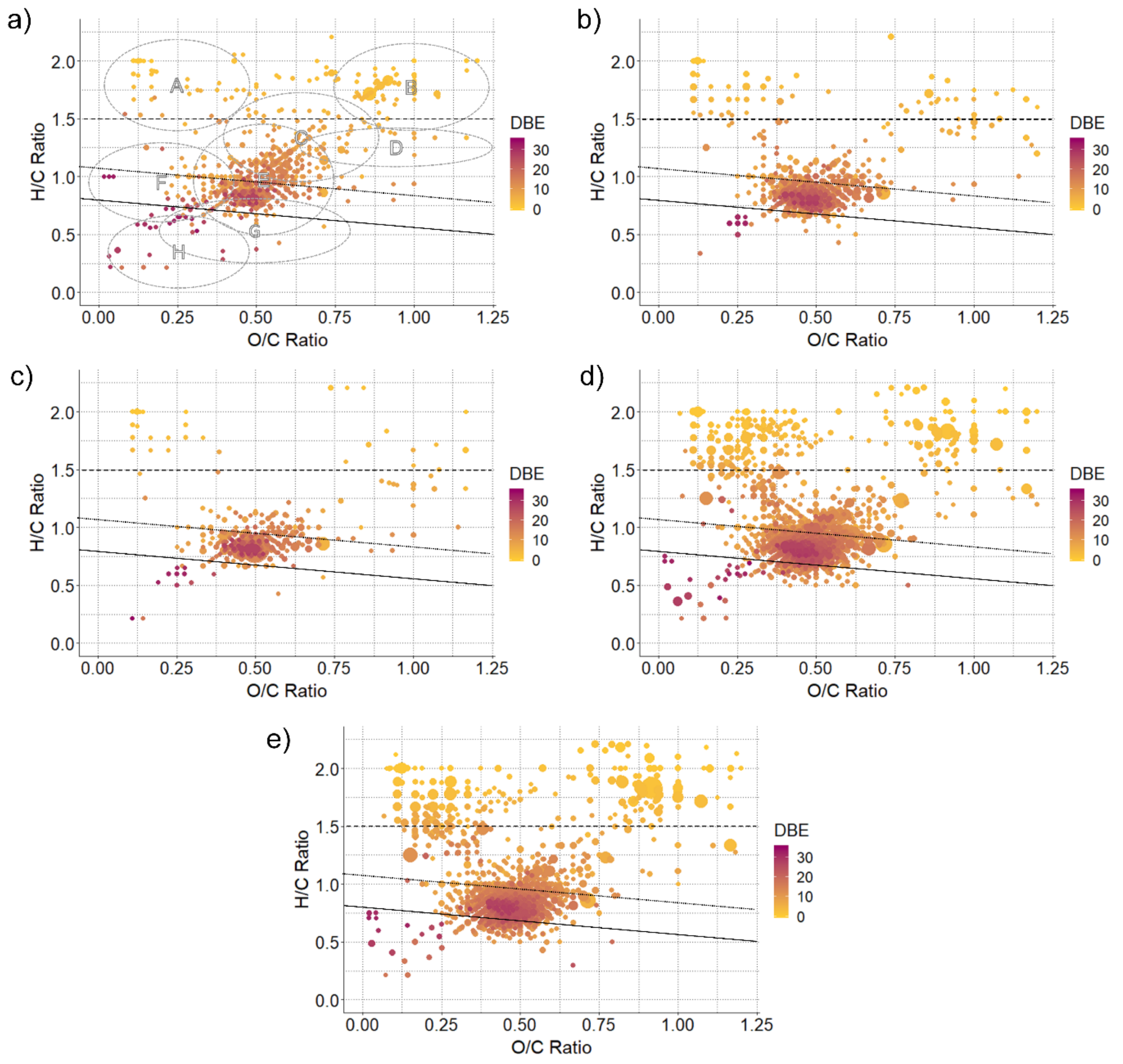

Fig. S31. Van Krevelen plots for representative samples whose NMR spectra are shown in Fig. 1. a) unburied, b) RMI, c) RMII, d) woodland and e) grassland. Points are color-coded by DBE. The size of points denotes abundance. The lines represent aliphatics (above the dashed line), aromatics (between the dotted and the full line) and condensed aromatics (below the full line) based on the AI_{mod} index. Letters denote the following compound classes, A: fatty acids/lipids, B: carbohydrates, C: glycosides, D: nucleic acids, E: polyphenols, F: lignin-like, G: oxidised polyphenols, H: condensed aromatics. The complexity and distribution of the types of compounds has changed upon burial. The unburied tea contains majority of the compounds in the 'polyphenol/glycoside' regions with others appearing in the 'carbohydrate' region. The woodland and grassland sites have greater diversity of compounds than unburied or peat bog buried green tea. While the 'aromatic/glycoside' region is still populated, both show increased number of compounds in the 'fatty acid/protein' and 'carbohydrate' regions. Comparing the peat bog sites, there appears to be identical types of compounds present, however in smaller numbers in RMII samples.

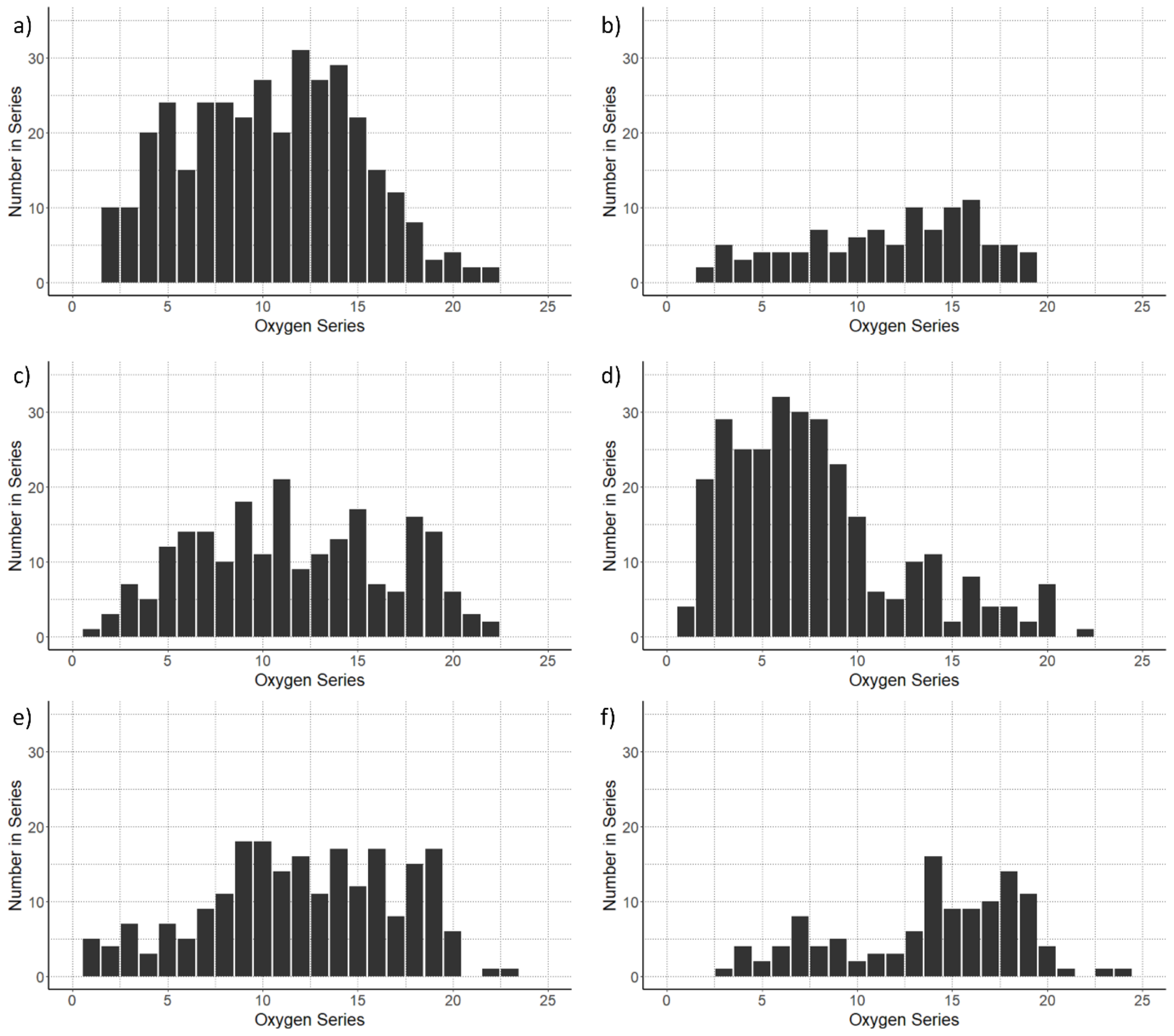

Fig. S32. Oxygen series for selected UpSet intersections a) preserved in all, I_2; b) produced in all, I_7; c) removed in all environments, I_5; d) produced in grassland only, I_4; e) produced in woodland only, I_6; f) produced in RMI or RMII, $I_{10}+I_{12}+I_{15}$.

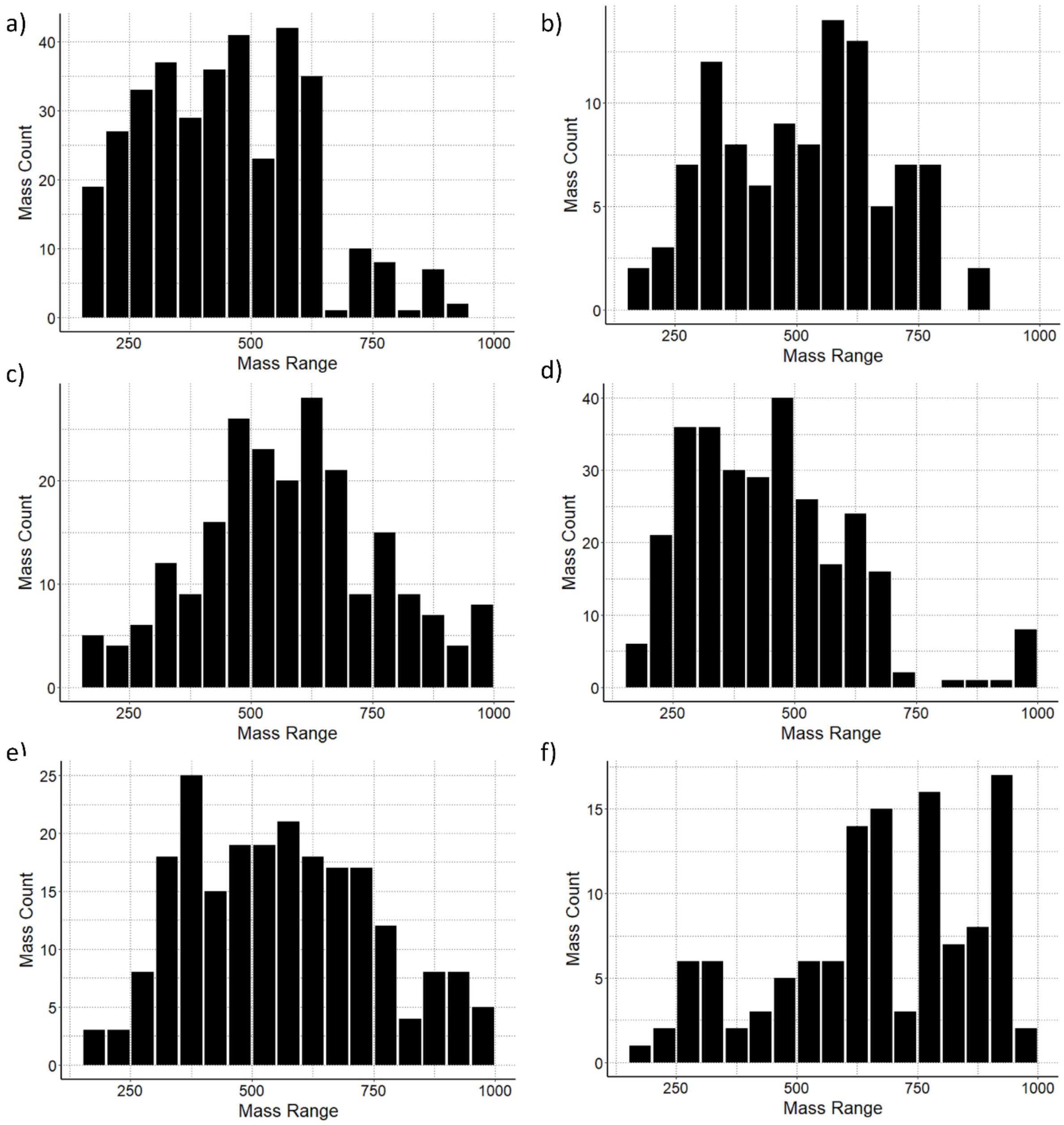

Fig. S33. Mass count for selected UpSet intersections a) preserved in all, I_2; b) produced in all, I_7; c) removed in all environments, I_5; d) produced in grassland only, I_4; e) produced in woodland only, I_6; f) produced in RMI or RMII, $I_{10}+I_{12}+I_{15}$.

Section 8. PCA of the FT-ICR-MS spectra of unburied and buried HWE green tea.

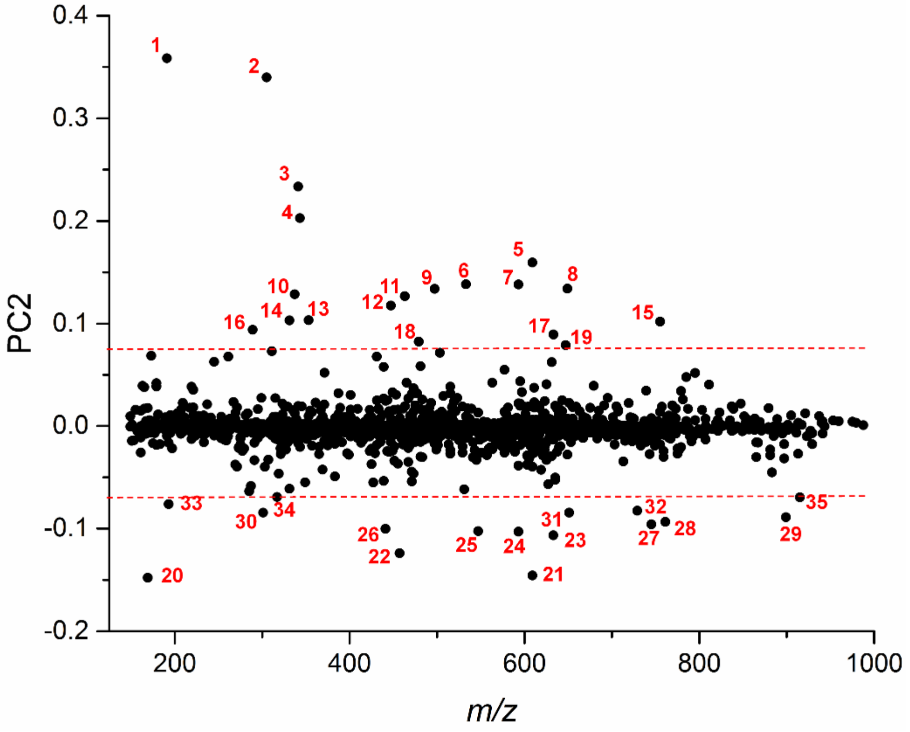

Fig. S34. PC2 loadings plot from the PCA of FT-ICR-MS spectra with unburied green tea. The compounds above and below the dotted lines are tentatively assigned based on literature.[4-10] **1.** $C_7H_{12}O_6$ (Quinic acid), **2.** $C_{22}H_{18}O_{10}$ (GC/ECG), **3.** $C_{12}H_{22}O_{11}$ (Sucrose), **4.** $C_{14}H_{16}O_{10}$ (Theogallin), **5.** $C_{27}H_{30}O_{16}$ (Rutin), **6.** $C_{19}H_{34}O_{17}$ (Sugar), **7.** $C_{27}H_{30}O_{15}$ (Quercetin glycoside derivative), **8.** $C_{22}H_{26}O_{13}$ (Glycoside), **9.** $C_{29}H_{30}O_{17}$ (Flavone), **10.** $C_{16}H_{18}O_8$ (p-coumaroylquinic acid), **11.** $C_{21}H_{20}O_{12}$ (Quercetin-3-*O*-galactoside/glucoside), **12.** $C_{21}H_{20}O_{11}$ (Cyanaroside), **13**. $C_{16}H_{18}O_9$ (Chlorogenic acid), **14.** $C_{13}H_{16}O_{10}$ (6-*O*-Galloyglucose), **15.** $C_{33}H_{40}O_{20}$ (Quercetin-3-*O*(glucosyl (1-3)rhamnosyl(1-6)rhamoside), **16.**$C_{15}H_{14}O_6$ (C/EC), **17.** $C_{29}H_{30}O_{16}$ (related to 9), **18.** $C_{21}H_{20}O_{13}$ (Myricetin 3'-glycoside/galactoside), **19**. $C_{27}H_{36}O_{18}$ (unknown), **20.** $C_7H_6O_5$ (Gallic Acid), **21**.$C_{29}H_{22}O_{15}$ (EGC digallate), **22.** $C_{22}H_{18}O_{11}$ (EGC/GC-3-*O*-gallate), **23.** $C_{27}H_{22}O_{18}$ (Ellagoyl-galloyl glucose), **24.** $C_{29}H_{22}O_{14}$ (EC/C 3,5-di-*O*-gallate), **25.** $C_{24}H_{20}O_{15}$ (), **26.** $C_{22}H_{18}O_{10}$ (EC/C gallate), **27.** $C_{37}H_{30}O_{17}$ (EGC-(4β->8)-EC-3-*O*-gallate ester), **28.** $C_{37}H_{30}O_{18}$ (Theasinensin B), **29.** $C_{44}H_{36}O_{21}$ (related to 34), **30.** $C_{15}H_{10}O_7$ (Quercetin), **31**. $C_{28}H_{28}O_{18}$ (Unknown), **32.** $C_{37}H_{30}O_{16}$ (Proanthocyanidin), **33.** $C_6H_{10}O_7$ (Glucuronic Acid), **34.** $C_{44}H_{36}O_{22}$ (Assamicain A), **35.** $C_{15}H_{10}O_8$ (Myricetin).

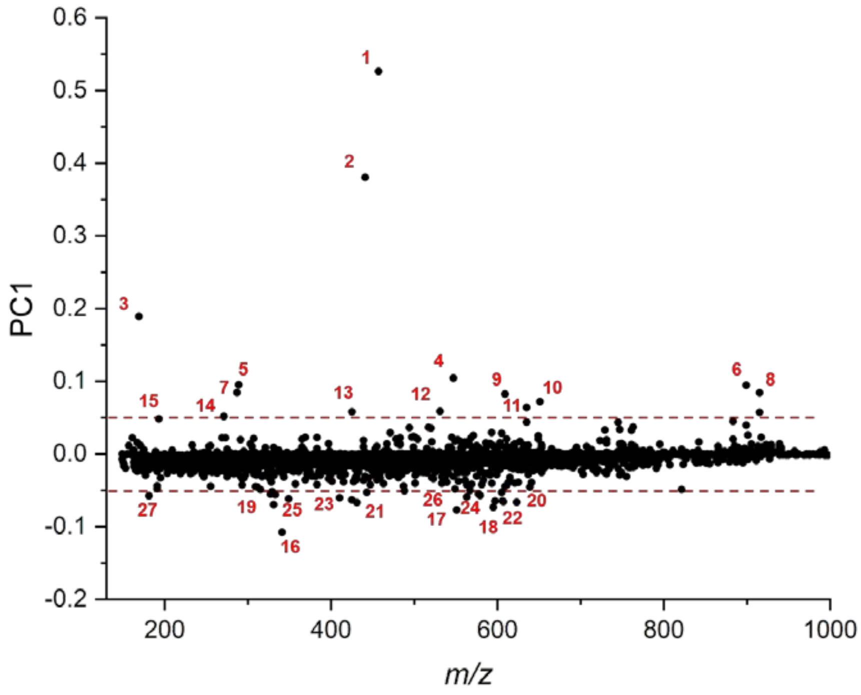

Fig. S35. PC1 loadings plot from the PCA of FT-ICR-MS spectra without unburied green tea. The compounds above and below the dotted lines are tentatively assigned based on literature.[4-10] **1. $C_{22}H_{18}O_{11}$ (EGCG)**, **2. $C_{22}H_{18}O_{10}$ (ECG)**, **3. $C_7H_6O_5$ (Gallic acid)**, **4.** $C_{24}H_{20}O_{15}$ (Unknown), **5.** $C_{15}H_{14}O_6$ (EC), **6.** $C_{44}H_{36}O_{21}$ (Related to 8), **7.** $C_{15}H_{12}O_6$ (2-Hydroxynaringenin), **8.** $C_{44}H_{36}O_{22}$ (Assamicain), **9.** $C_{29}H_{22}O_{15}$ (EGC- 5, 7,-di-*O*-gallate), **10.** $C_{28}H_{28}O_{18}$ (Unknown), **11.**$C_{27}H_{24}O_{18}$ (Trigalloyl glucose), **12.** $C_{24}H_{20}O_{14}$ (Related to 4), **13.** $C_{22}H_{18}O_9$ (Epiafzelechin 3-*O*-gallate), **14.** $C_{15}H_{12}O_5$ (Naringenin), **15.** $C_6H_{10}O_7$ (Glucuronic acid), **16. $C_{12}H_{22}O_{11}$ (Trehalose)**, **17.** $C_{28}H_{24}O_{12}$ (Unknown), **18.**$C_{29}H_{24}O_{14}$ (Related to Thea flavin), **19.** $C_{13}H_{16}O_{10}$ (6-*O*-Galloylglucose), **20.**$C_{30}H_{24}O_{15}$ (Related to 23)**, 21. $C_{14}H_{24}O_{15}$ (Sugar)**, **22.** $C_{30}H_{24}O_{14}$ (Theacitrin), **23.** $C_{21}H_{14}O_{10}$ (Unknown), **24.** $C_{29}H_{26}O_{14}$ (Unknown), **25. $C_{16}H_{14}O_9$ (Aromatic)**, **26.** $C_{26}H_{28}O_{14}$ (Apiin), **27.** $C_6H_{14}O_6$ (Mannitol). Formulae/compounds shown in bold made the largest contribution to the definition of the PCA-DA shown in Fig. S37

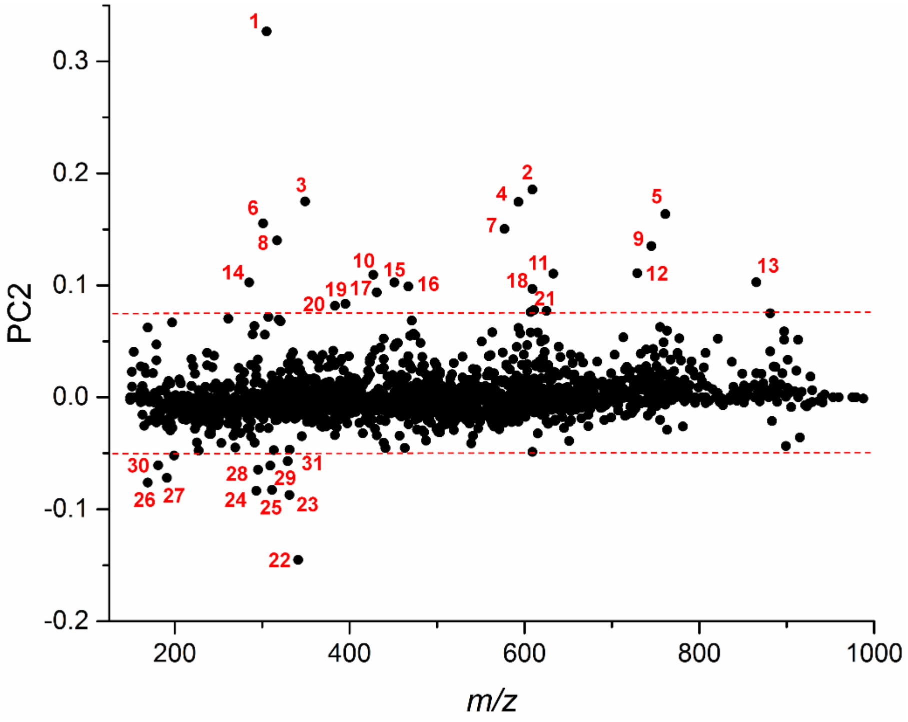

Fig. S36. PC2 loadings plot from the PCA of FT-ICR-MS spectra without unburied green tea. The compounds above and below the dotted lines are tentatively assigned based on literature.[4-10] **1. $C_{15}H_{14}O_7$ (GC)**, **2. $C_{30}H_{26}O_{14}$ (Theasinensin C)**, **3. $C_{16}H_{14}O_9$ (Aromatic)**, **4. $C_{30}H_{26}O_{13}$ (Catechin dimer)**, **5. $C_{37}H_{30}O_{18}$ (Theasinensin B)**, **6. $C_{15}H_{10}O_7$ (Quercetin)**, **7.** $C_{30}H_{26}O_{12}$ (Procyanidin B3), **8. $C_{15}H_{10}O_8$ (Myricetin)**, **9. $C_{37}H_{30}O_{17}$ (EGC-EC gallate)**, **10. $C_{21}H_{16}O_{10}$ (Theaflavic acid)**, **11. $C_{27}H_{22}O_{18}$ (Strictinin)**, **12. $C_{37}H_{30}O_{16}$ (EGC-(4β→8)-EC-3-*O*-gallate ester)**, **13. $C_{45}H_{38}O_{18}$ (Procyanidin C1)**, **14.** $C_{15}H_{10}O_6$ (Kaempferol), **15.** $C_{24}H_{20}O_9$ (EGC 3-O-p-coumarate), **16.** $C_{24}H_{20}O_{10}$ (EGC 3-O-caffeate), **17.** $C_{21}H_{20}O_{10}$ (Vitexin), **18.** $C_{29}H_{22}O_{15}$ (EGC 3, 4',-di-O-gallate), 1**9.** $C_{17}H_{16}O_{11}$ (aromatic), **20.** $C_{14}H_{24}O_{12}$ (O-Acetylsucrose), **21.** $C_{30}H_{24}O_{14}$ (Prodelphinidin A1),), **23.** $C_{13}H_{10}O_{10}$ (6-O-galloyl-β-D-glucose, **22. $C_{12}H_{22}O_{11}$ (Trehalose)**, **24.** $C_{18}H_{30}O_3$ (Fatty acid), **25.** $C_{18}H_{32}O_4$ (Fatty acid), **26.** $C_7H_6O_5$ (Gallic acid), **27.** $C_6H_8O_7$ (Citric acid), **28.** $C_{18}H_{32}O_3$ (Fatty acid), **29.** $C_{22}H_{18}O_{10}$ (ECG/CG), **30.** $C_{18}H_{30}O_4$ (Fatty acid), **31.** $C_6H_{14}O_6$ (Mannitol). Formulae shown in bold made the largest contribution to the definition of the PCA-DA shown in Fig. S37

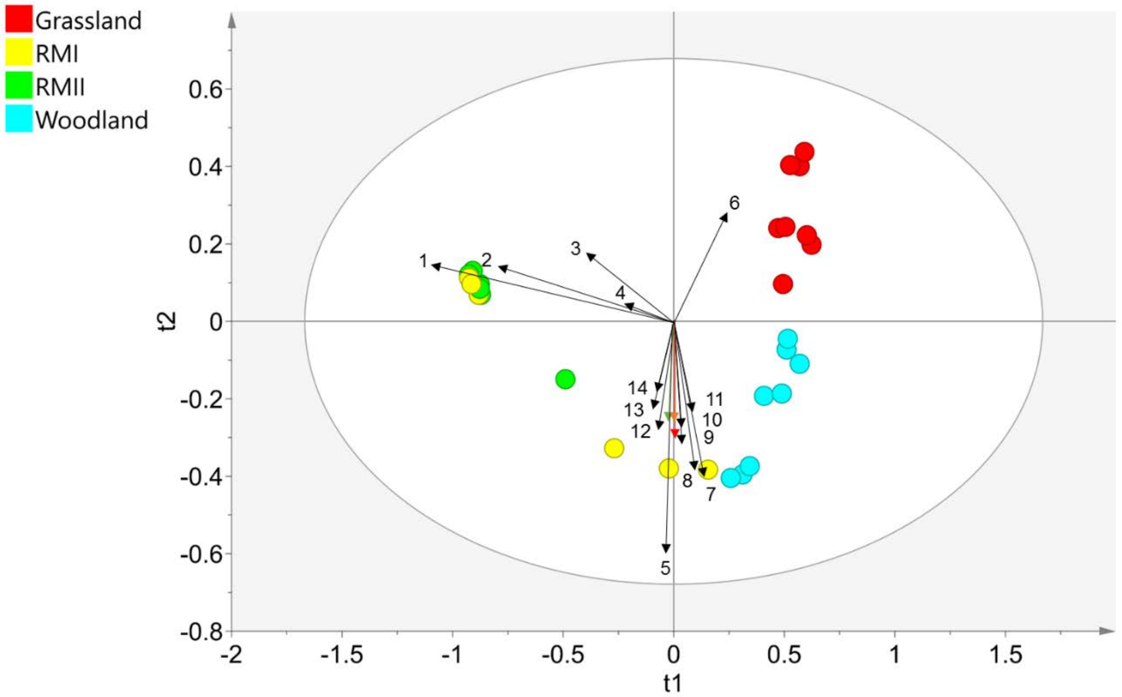

Fig. S37. PLS-DA score plot based on the MS spectra of the HWE buried green tea samples without unburied green tea ($R^2X = 0.538$, $R^2Y = 0.317$ and $Q^2 = 0.317$). The Hotelling's T^2 ellipse represents 95% confidence interval. The key indicates the colour coding for each sample site. Overlaid biplots indicate the loadings of major discriminatory compounds along PC1 and PC2 dimension. **1.** EGCG, **2.** ECG/CG, **3.** Gallic acid, **4.** $C_{24}H_{20}O_{15}$, **5.** EGC/GC, **6.** Trehalose, **7.** $C_{16}H_{14}O_9$, **8.** Theasinensin C, **9.** $C_{30}H_{26}O_{13}$ (catechin dimer), **10.** Procyanidin C1, **11.** Theaflavic acid, **12.** Theasinensin B, **13.** EGC/GC-3-*O*-gallate, **14.** $C_{37}H_{30}O_{17}$ (EGC-(4β->8)-EC-3-*O*-gallate ester), Strictinin (green), Myricetin (oranga), Quercetin (red).

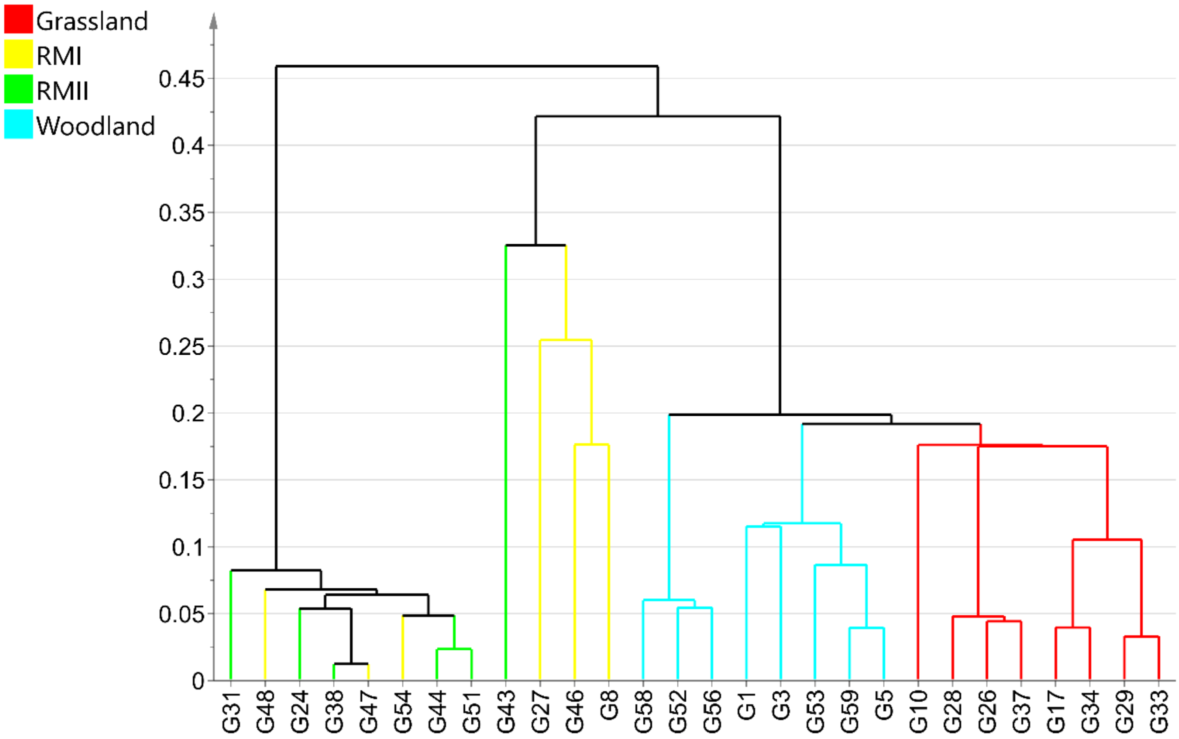

Fig. S38. HCA dendrogram based on the PLS-DA of MS spectra of the HWE buried green tea samples without unburied green tea. The key indicates the colour coding for each sample site.

References

1 Robinson, P. T., Pham, T. N. & Uhrin, D. In phase selective excitation of overlapping multiplets by gradient-enhanced chemical shift selective filters. *Journal of Magnetic Resonance* **170**, 97-103, doi:10.1016/j.jmr.2004.06.004 (2004).

2 Hansen, P. I. *et al.* Structure and Hydration of the Amylopectin Trisaccharide Building Blocks-Synthesis, NMR, and Molecular Dynamics. *Biopolymers* **89**, 1179-1193, doi:10.1002/bip.21075 (2008).

3 Evans, R. *et al.* Quantitative Interpretation of Diffusion-Ordered NMR Spectra: Can We Rationalize Small Molecule Diffusion Coefficients? *Angewandte Chemie-International Edition* **52**, 3199-3202, doi:10.1002/anie.201207403 (2013).

4 Lee, L. S., Kim, S. H., Kim, Y. B. & Kim, Y. C. Quantitative Analysis of Major Constituents in Green Tea with Different Plucking Periods and Their Antioxidant Activity. *Molecules* **19**, 9173-9186, doi:10.3390/molecules19079173 (2014).

5 Lee, J. E. *et al.* H-1 NMR-based metabolomic characterization during green tea (Camellia sinensis) fermentation. *Food Research International* **44**, 597-604, doi:10.1016/j.foodres.2010.12.004 (2011).

6 van der Hooft, J. J. J. *et al.* Structural Annotation and Elucidation of Conjugated Phenolic Compounds in Black, Green, and White Tea Extracts. *Journal of Agricultural and Food Chemistry* **60**, 8841-8850, doi:10.1021/jf300297y (2012).

7 Le Gall, G., Colquhoun, I. J. & Defernez, M. Metabolite profiling using H-1 NMR spectroscopy for quality assessment of green tea, Camellia sinensis (L.). *Journal of Agricultural and Food Chemistry* **52**, 692-700, doi:10.1021/jf034828r (2004).

8 Xin, Z. Q. *et al.* UPLC-Orbitrap-MS/MS combined with chemometrics establishes variations in chemical components in green tea from Yunnan and Hunan origins. *Food Chemistry* **266**, 534-544, doi:10.1016/j.foodchem.2018.06.056 (2018).

9 Kuhnert, N., Drynan, J. W., Obuchowicz, J., Clifford, M. N. & Witt, M. Mass spectrometric characterization of black tea thearubigins leading to an oxidative cascade hypothesis for thearubigin formation. *Rapid Communications in Mass Spectrometry* **24**, 3387-3404, doi:10.1002/rcm.4778 (2010).

10 Sharma, R., Gogna, N., Singh, H. & Dorai, K. Fast profiling of metabolite mixtures using chemometric analysis of a speeded-up 2D heteronuclear correlation NMR experiment. *Rsc Advances* **7**, 29860-29870, doi:10.1039/c7ra04032f (2017).
